# Blocking the angiopoietin-2–dependent integrin **β**-1 signaling axis abrogates small cell lung cancer invasion and metastasis

**DOI:** 10.1172/jci.insight.166402

**Published:** 2024-05-22

**Authors:** Lydia Meder, Charlotte Isabelle Orschel, Christoph Julius Otto, Mirjam Koker, Johannes Brägelmann, Meryem S. Ercanoglu, Sabrina Dähling, Anik Compes, Carolin Selenz, Marieke Nill, Felix Dietlein, Alexandra Florin, Marie-Lisa Eich, Sven Borchmann, Margarete Odenthal, Raquel Blazquez, Frank Hilberg, Florian Klein, Michael Hallek, Reinhard Büttner, H. Christian Reinhardt, Roland T. Ullrich

**Affiliations:** 1 Friedrich-Alexander-Universität Erlangen-Nürnberg, Faculty of Medicine, Department of Experimental Medicine 1, Erlangen, Germany.; 2 Mildred Scheel School of Oncology and; 3 Department I of Internal Medicine, University of Cologne, Faculty of Medicine at the University Hospital Cologne, Cologne, Germany.; 4 Center for Molecular Medicine Cologne, University of Cologne, Cologne, Germany.; 5 Department of Translational Genomics and; 6 Institute of Virology, Laboratory of Experimental Immunology, University of Cologne, Faculty of Medicine at the University Hospital Cologne, Cologne, Germany.; 7 Department of Medical Oncology, Dana-Faber Cancer Institute, Boston, Massachusetts, USA.; 8 Cancer Program, Broad Institute of MIT and Harvard, Cambridge, Massachusetts, USA.; 9 Institute for Pathology, University of Cologne, Faculty of Medicine at the University Hospital Cologne, Cologne, Germany.; 10 Else Kröner Forschungskolleg Clonal Evolution in Cancer, University Hospital Cologne, Cologne, Germany.; 11 German Hodgkin Study Group, Department I of Internal Medicine, University Hospital Cologne, Cologne, Germany.; 12 Center for Integrated Oncology Aachen Bonn Cologne Duesseldorf, University of Cologne, Faculty of Medicine at the University Hospital Cologne, Cologne, Germany.; 13 University Hospital Regensburg, Department of Internal Medicine III, Hematology and Medical Oncology, Regensburg, Germany.; 14 Department of Pharmacology, Boehringer Ingelheim RCV GmbH & Co KG, Vienna, Austria.; 15 Department of Hematology and Stem Cell Transplantation, West German Cancer Center, University Hospital Essen, German Cancer Consortium (DKTK), Essen, Germany.

**Keywords:** Oncology, Lung cancer

## Abstract

Small cell lung cancer (SCLC) is the most aggressive lung cancer entity with an extremely limited therapeutic outcome. Most patients are diagnosed at an extensive stage. However, the molecular mechanisms driving SCLC invasion and metastasis remain largely elusive. We used an autochthonous SCLC mouse model and matched samples from patients with primary and metastatic SCLC to investigate the molecular characteristics of tumor metastasis. We demonstrate that tumor cell invasion and liver metastasis in SCLC are triggered by an Angiopoietin-2 (ANG-2)/Integrin β-1–dependent pathway in tumor cells, mediated by focal adhesion kinase/Src kinase signaling. Strikingly, CRISPR-Cas9 KO of Integrin β-1 or blocking Integrin β-1 signaling by an anti–ANG-2 treatment abrogates liver metastasis formation in vivo. Interestingly, analysis of a unique collection of matched samples from patients with primary and metastatic SCLC confirmed a strong increase of Integrin β-1 in liver metastasis in comparison with the primary tumor. We further show that ANG-2 blockade combined with PD-1–targeted by anti-PD-1 treatment displays synergistic treatment effects in SCLC. Together, our data demonstrate a fundamental role of ANG-2/Integrin β-1 signaling in SCLC cells for tumor cell invasion and liver metastasis and provide a potentially new effective treatment strategy for patients with SCLC.

## Abstract 2

The angiopoeitin-2&ndash;dependent integrin &beta;-1 signaling pathway regulates small cell lung carcinoma invasion and metastasis and represents a potential therapeutic target.

## Graphical abstract

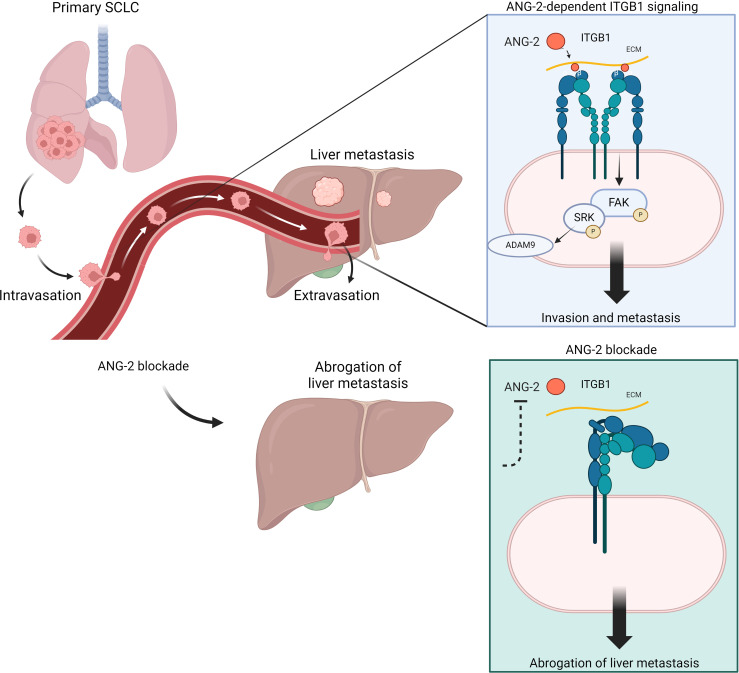

## Introduction

Small cell lung carcinomas (SCLC) account for ~30,000 deaths in the United States per year and represent, with ~15% of cases, the most aggressive form of lung cancer with early systemic metastasis. SCLC is characterized by fast tumor growth with very poor outcome due to rapid recurrence after standard chemotherapy and early formation of metastasis (1). Increased chromatin accessibility was described as the genetic reason for the prometastatic neuronal gene expression program of SCLC (2). However, so far, the molecular mechanism that induces tumor cell invasion and formation of metastasis remains largely elusive (3).

It is described that solid tumors modulate the extracellular matrix (ECM) to create a microenvironment that fosters tumorigenesis and metastasis (4). Integrins are cell-surface receptors that interact with the ECM and regulate processes important for tumor establishment, maintenance, and progression such as proliferation, differentiation, migration and apoptosis (5). Integrins form membrane-bound heterodimers consist of an α and a β subunit that enable outside-in and inside-out signaling upon activation. Outside-in integrin signals are not only provided by ECM proteins but also by soluble ligands like Angiopoietin-2 (ANG-2) (6, 7).

Previous studies particularly suggest integrin β-1 (ITGB1) as marker of poor prognosis in SCLC (8, 9). It has been shown that SCLC primary and metastatic lesions are embedded in dense ECM, where ITGB1 contributes to SCLC chemotherapy resistance (10).

Overall, there is growing evidence that ITGB1 plays an important role in SCLC progression and metastasis via a previously unknown mechanism. In this study, we unravel the ITGB1-dependent signaling axis in SCLC and provide a potentially novel therapeutic approach using BI-836880, a bispecific inhibitor of ANG-2/VEGF-A. BI 836880 works bispecifically in humans but only against ANG-2 in mice (11). Thus, combining BI-836880 with anti–PD-1 and VEGFR inhibition in an autochthonous mouse model of SCLC is potent to combat SCLC metastasis.

## Results

### ITGB1 expression significantly correlates with tumor stage in patients and is increased in matched SCLC liver metastases.

**Figure 1:**
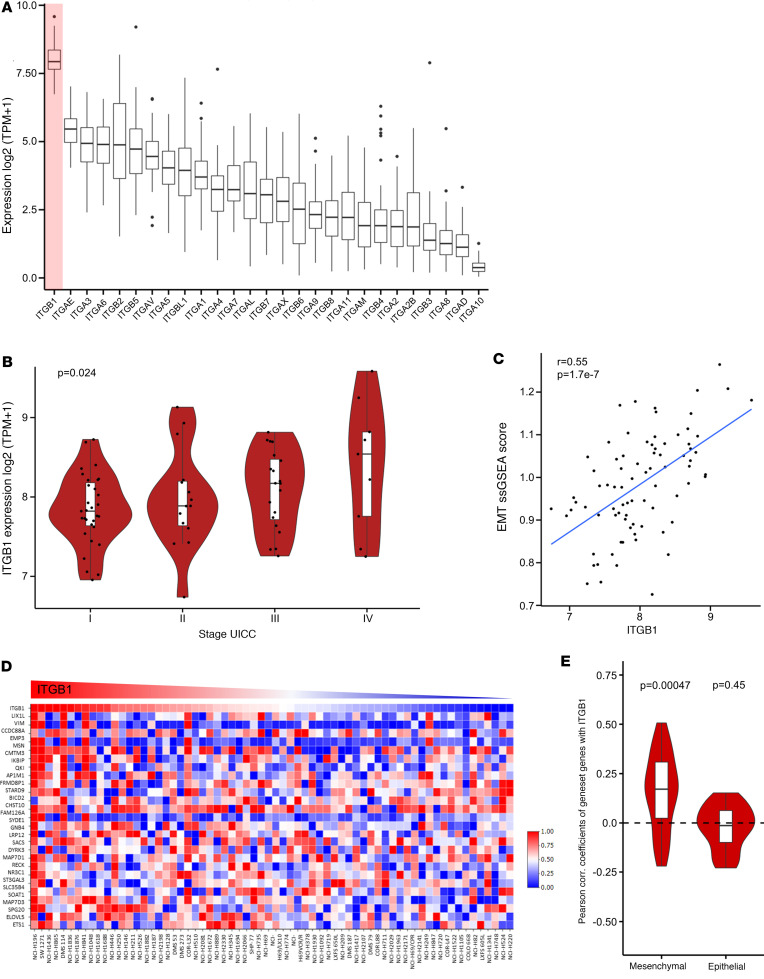
ITGB1 correlates with late tumor stage and EMT in patients. Human SCLC data were analyzed regarding ITGB1 correlations. (**A**) RNA-Seq of human SCLC material was analyzed for the expression of integrin genes (*n* = 81). (**B**) Association between ITGB1 expression and UICC tumor stage (*n* = 75) were tested using the nonparametric Jonckheere-Terpstra test. (**C**) A single-sample Gene Set Enrichment Analysis (ssGSEA) was performed using the “GSVA” R package against the MSigDB Hallmark EMT gene set from which ITGB1 was removed. (**D**) mRNA expression data for SCLC provided the “mesenchymal” gene set in human SCLC cell lines (*n* = 64) that was correlated to ITGB1 gene expression. The heatmap was generated by the heatmap tool provided by SCLC-CellMiner. (**E**) Pearson correlation coefficients with ITGB1 expression in human SCLC cell lines of all genes per “mesenchymal” and “epithelial” gene set (*P* values from 1-sample Wilcoxon-tests for median correlation coefficient equal to zero per gene set). Data of mRNA gene expression are listed in the Supplemental Table 1.

Since SCLC represents the most aggressive type of lung cancer, with frequent systemic metastasis (3), we aimed to investigate the effect of integrins on metastasis formation in SCLC. We analyzed RNA-Seq data sets of patients with SCLC (12) and identified ITGB1 as the integrin with the highest expression (Figure 1A). Of note, ITGB1 significantly correlated with advanced UICC tumor stage and epithelial-to-mesenchymal transition (EMT) (Figure 1, B and C), which is highly dynamic and frequently observed in progressing tumors (13). Other integrins, such as ITGB3, ITGB5, and ITGA1, positively correlated with tumor stage in SCLC (Supplemental Figure 1; supplemental material available online with this article; https://doi.org/10.1172/jci.insight.166402DS1). Next, we analyzed transcriptomic data from human SCLC cell lines (14). mRNA gene expression data from 64 SCLC cell lines (15) revealed that ITGB1 expression significantly correlated with “mesenchymal” gene expression compared with “epithelial” gene expression (Figure 1, D and E, and Supplemental Table 1) and with “migration” gene expression (Supplemental Figure 2 and Supplemental Table 1). On the protein level, SCLC cell lines obtained from metastatic sites displayed a higher ITGB1 expression than cell lines obtained from the primary tumor site of the lung (Supplemental Figure 3).

**Figure 2:**
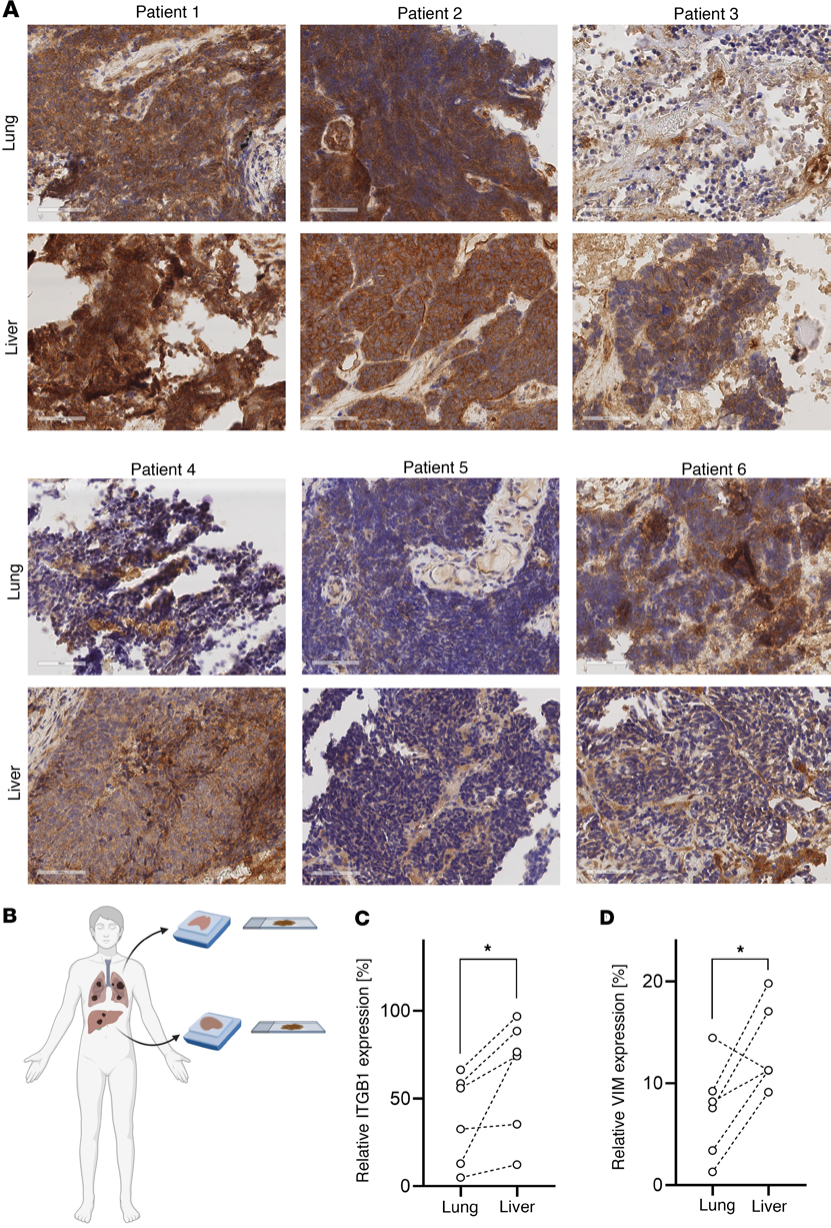
ITGB1 is increased in liver metastasis of patients with SCLC. (**A**) FFPE lung tumor material of 6 patients with SCLC and matched liver metastasis was analyzed for the expression of ITGB1. (**B**) Schematic of matched SCLC patient samples obtained from a primary lung tumor and liver metastasis. Created with BioRender.com. (**C** and **D**) IHC stained slides were scanned and analyzed regarding ITBG1 expression and Vimentin expression using ImageScope and ImageJ. Representative images of ITGB1 for each patient (*n* = 6) are shown in **A** and for Vimentin in Supplemental Figure 4. Statistical analysis was done using 2 way ANOVA (*P < 0.05; data are shown as mean ± SEM). Clinicopathologic characteristics are listed in the Supplemental Table 2. Scale bars: 60 μm.

In addition, we analyzed a unique collection of tumor tissue from 6 patients with SCLC with matched samples from primary SCLC lung and metastatic SCLC liver tumors. In line with our previous findings, we detected increased ITGB1 expression and increased expression of the mesenchymal marker Vimentin in matched liver metastasis compared with the primary SCLC (Figure 2, Supplemental Figure 4, and Supplemental Table 2). Thus, the clinical data further support our hypothesis that integrins, particularly ITGB1, are important factors in the metastatic cascade of SCLC.

### ITGB1 is increased on SCLC cells upon extensive stage of disease in mice and drives tumor cell migration.

**Figure 3:**
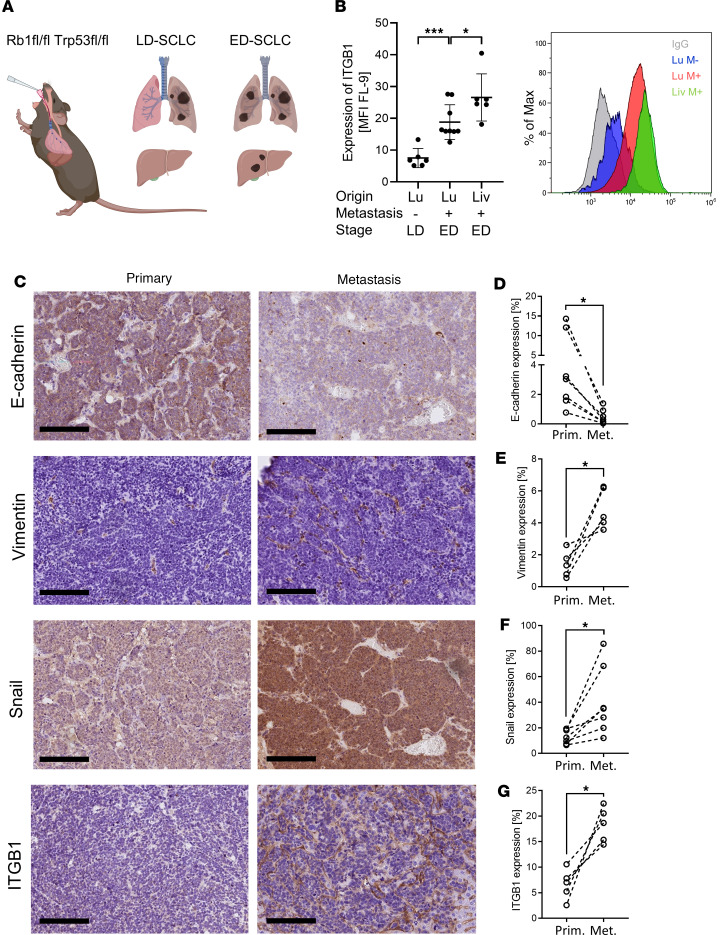
ITGB1 is increased on tumor cells upon extensive stage of disease and correlates with EMT in an autochthonous SCLC mouse model. SCLC-bearing mice were analyzed for metastatic disease and the expression of the ANG-2 receptor ITGB1. (**A**) Schematic of the autochthonous Rb1-loss– and Trp53-loss–driven SCLC mouse model developing limited stage of disease (LD-SCLC) and extensive stage of disease (ED-SCLC). Created with BioRender.com. (**B**) Relative expression of ITGB1 on SCLC tumor cells isolated from mice (*n* = 6–9) normalized to IgG control, determined by flow cytometry. Origin of tumor cells, presence of metastasis, and stage are indicated. (**C**) Representative IHC results for E-cadherin, Vimentin, Snail, and ITGB1 in matched murine tumors obtained from the primary SCLC and SCLC liver metastasis. Scale bars: 200 μm. (**D**–**G**) Quantification of protein expression (*n* = 5–7) using the threshold method of ImageJ in matched samples. Statistical analysis was done using 2 way ANOVA (*P < 0.05; **P < 0.01; ***P < 0.001; data are shown as mean ± SEM).

**Figure 4:**
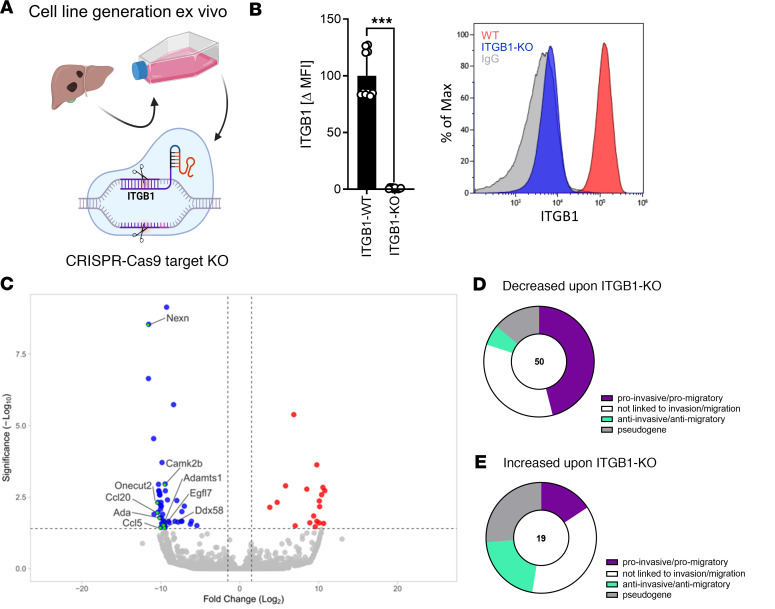
ITGB1-KO reduced the promigratory and proinvasive gene signatures in SCLC. (**A**) Schematic of ITGB1-KO cell line generation obtained from murine liver metastasis. Created with BioRender.com. (**B**) Relative expression of the ITGB1 SCLC tumor cells before and after ITGB1-KO as mean fluorescence intensity (MFI) normalized to IgG control, determined by flow cytometry with representative histograms. Statistical analysis was done using 2 way ANOVA (***P < 0.001; data are shown as mean ± SEM). (**C**) Volcano plot of RNA-Seq data of ITGB1-KO versus controls (*n* = 3 per group). DEGs downregulated upon ITGB1-KO are depicted in blue, and those upregulated are depicted in red. GO enrichment analysis listed in Supplemental Table 4 revealed genes of the pathway GO:0030334 (regulation of cell migration) enriched, indicated in green. (**D** and **E**) Significantly deregulated genes were classified as proinvasive/promigratory, not linked to invasion/migration, antiinvasive/antimigratory, or pseudogene listed in Supplemental Table 3 and compared regarding their enrichment upon ITGB1-KO.

We next aimed to validate our clinical findings in an autochthonous SCLC mouse model, in which tumors are induced upon Cre-mediated biallelic deletion of *Rb1* and *Tp53* (16). We compared mice with limited stage of disease (LD) with mice with extensive stage of disease (ED) (Figure 3A) regarding their ITGB1 expression and markers for EMT. Flow cytometry analysis of surface ITGB1 indicated a significant upregulation of ITGB1 upon disease progression on SCLC cells obtained from liver metastasis (Figure 3B). In contrast, ITGB3 and ITGA5 — which have been associated to high tumor stages in patients (Supplemental Figure 1) — are not upregulated upon liver metastasis formation in vivo (Supplemental Figure 5, A and B). Also TIE-2, a frequently described receptor of ANG-2 (17), was not upregulated upon metastasis formation (Supplemental Figure 5, C–E). Strikingly, the matched SCLC liver metastasis in mice mimicked the EMT features found in SCLC patient material with significantly decreased E-cadherin and significantly increased Vimentin and Snail in liver metastases compared with matched primary lung tumors (Figure 3, C–F). Moreover, higher ITGB1 expression in metastases compared with the primary lung tumors had been validated based on IHC analysis, whereas ANG-2 is similarly expressed in primary tumors and liver metastasis (Figure 3, C and G, and Supplemental Figure 6). To explore ITGB1 regulated gene signatures, we knocked out ITGB1 by Crispr-Cas9 (Figure 4, A and B). RNA-Seq analysis and gene ontology (GO) enrichment analysis revealed that ITGB1-KO induced downregulation of proinvasive and promigratory genes and upregulation of antiinvasive and antimigratory genes (Figure 4, C–E, and Supplemental Tables 3 and 4).

**Figure 5:**
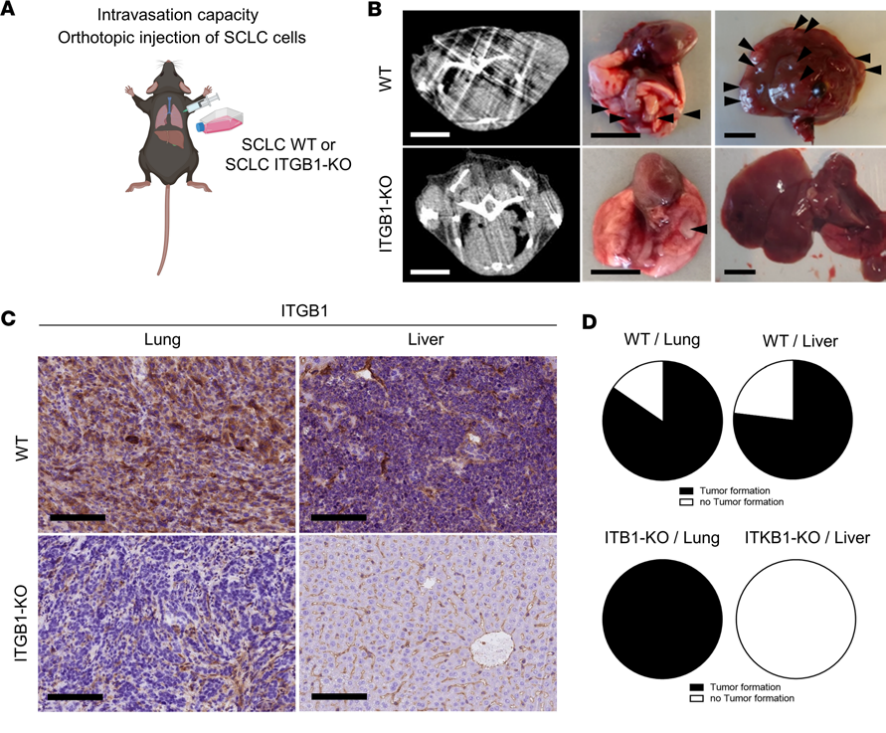
ITGB1 signaling is needed for intravasation of SCLC cells. (**A**) Schematic of orthotopic injection of SCLC cells to determine intravasation capacity. Created with BioRender.com. (**B**) Representative μCT of WT SCLC clones and ITGB1-KO injected into the lungs and growing in vivo with representative images of murine lungs and liver postmortem (*n* = 5 per group). (**C**) Representative images of ITGB1 IHC stain of SCLC WT and ITGB1-KO orthotopically injected into the lung (*n* = 5 per group). Scale bars: 100 μm. (**D**) The capability of WT SCLC clones and ITGB1-KO to form tumors after orthotopic injection was determined by IHC based on H&E and NCAM and quantified for lungs and livers, respectively (*n* = 5).

To validate ITGB1 as a key mediator of SCLC migration and invasion, we carried out an in vivo approach with different routes of tumor cell injection, either orthotopically into the lungs and i.v. to explore ITGB1-KO effects on intra- and extravasation capacity of tumor cells. Orthotopic injection of tumor cells resulted in measurable target lesions in the lungs after 2 weeks (Figure 5, A and B). Harvested tumors indicated ITGB1 expression in SCLC WT and ITGB1 depletion in SCLC ITGB1-KO (Figure 5C). In addition, harvested lungs and livers were analyzed regarding the detection of tumor lesions based on H&E and neural cell adhesion molecule (NCAM) (Supplemental Figure 7A). Upon ITGB1-KO, no liver metastases were detected, whereas 100% of injections resulted in lung tumor growth (Figure 5D), reflecting the lack of intravasation capacity of ITGB1-KO. In a next step, we injected ITGB1-KO and WT controls i.v. to determine their extravasation capacity. Neither in the lung nor in the liver, SCLC cells were able to form lesions upon ITGB1-KO (Supplemental Figure 7, B and C). Together, we showed that ITGB1 is indispensable for intravasation and extravasation of SCLC. Consequently, our in vivo and in vitro data confirm a pivotal role of ITGB1 in SCLC liver metastasis.

### ITGB1 signaling blockade abrogates SCLC liver metastases in vivo.

ANG-2 has been shown to mediate migration by ITGB1 signaling (18, 19). Thus, we investigated the effect of ANG-2–mediated stimulation of ITGB1 on SCLC cell migration and the formation of liver metastasis.

**Figure 6:**
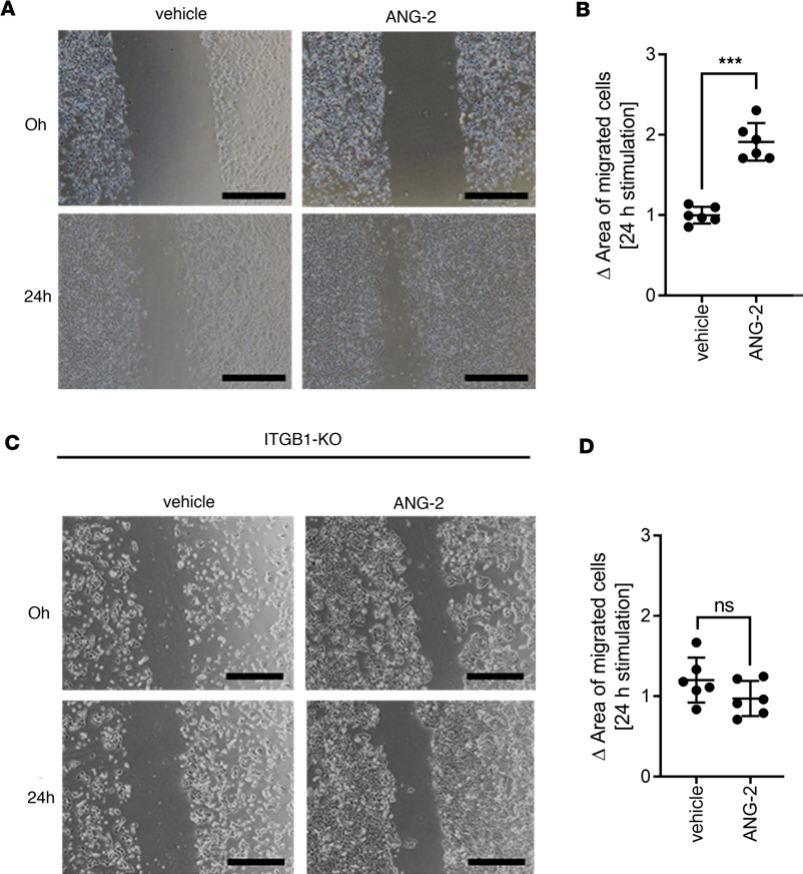
ANG-2–dependent ITGB1 signaling triggers SCLC cell migration. (**A** and **B**) The area of migrated SCLC tumor cells isolated from liver metastasis was determined by scratch assay (*n* = 6). Cells were stimulated with ANG-2 for 24 hours. Images after 0 and 24 hours were analyzed using ImageJ. Statistical analysis was done using the Student’s 2-tailed *t* test (*P < 0.05; **P < 0.01; ***P < 0.001; data are shown as mean ± SEM). (**C** and **D**) The area of migrated SCLC tumor cells with Crispr ITGB1-KO was determined by scratch assay (*n* = 6). Cells were stimulated with ANG-2 for 24 hours. Statistical analysis was done using the 2-tailed Student’s *t* test (*P < 0.05; **P < 0.01; ***P < 0.001; data are shown as mean ± SEM).

**Figure 7:**
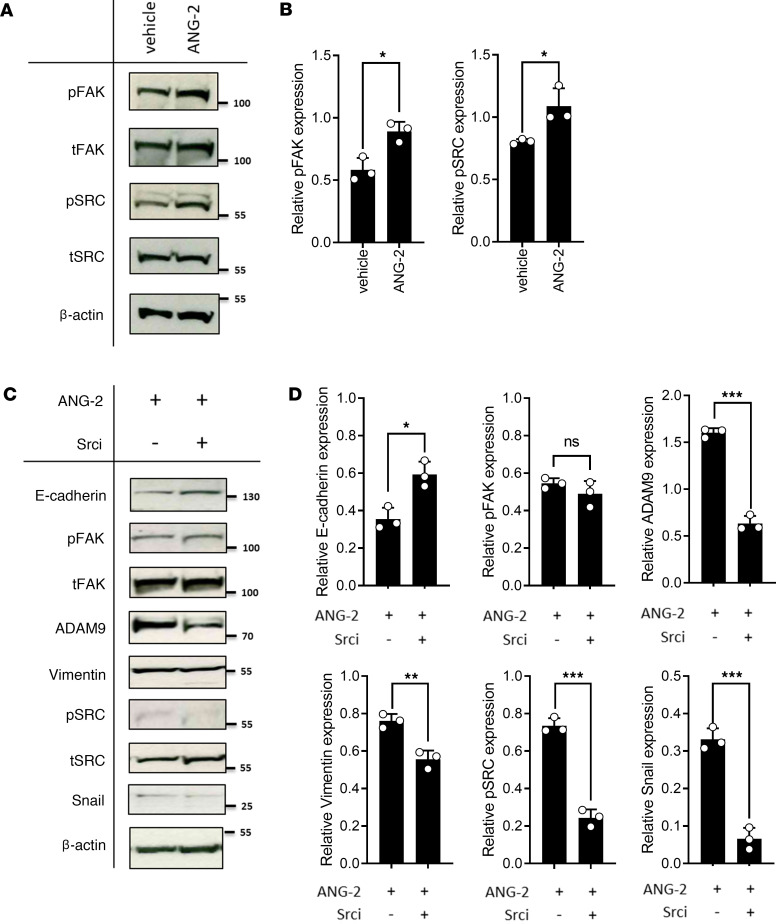
ANG-2–dependent ITGB1 signaling triggers FAK-SRC signaling in SCLC. (**A**) Intracellular FAK-SRC signaling was determined by Western blot upon ANG-2 stimulation of cultured SCLC cells isolated from liver metastasis. Representative experiment out of 3. (**B**) Western blots were quantified according to total protein levels and normalized to β-actin. Statistical analysis was done using the 2-tailed Student’s *t* test (*P < 0.05; **P < 0.01; ***P < 0.001; data are shown as mean ± SEM). (**C**) EMT proteins, components of the FAK-SRC signaling were determined by Western blot upon ANG-2 stimulation and simultaneous treatment by 100 nM Saracatinib. Representative experiment out of 3. (**D**) Western blots were quantified according to total protein levels and normalized to β-actin. Statistical analysis was done using the 2-tailed Student’s *t* test (*P < 0.05; **P < 0.01; ***P < 0.001; data are shown as mean ± SEM).

In vitro assays depicted a significant increase of migration after ANG-2 stimulation (Figure 6, A and B), which is diminished upon ITGB1-KO (Figure 6, C and D). Src inhibition by Saracatinib abrogated ANG-2–dependent SCLC cell migration and intracellular signaling in a similar manner as ITGB1-KO (Figure 7 and Supplemental Figure 8), and Src and Fak showed increased phosphorylation upon ANG-2 stimulation at Tyr-416 and Tyr-397, respectively (Figure 7, A and B). This indicates that ANG-2– and ITGB1-mediated signaling acts upstream of the prominent Src/Fak-driven pathway in SCLC cell migration.

We further show that stimulation of ITGB1 with ANG-2 and fibronectin showed a similar RNA-Seq profile of migratory gene regulation in SCLC (Supplemental Figure 9A). For example, ADAM9 was upregulated upon ITGB1 stimulation and downregulated upon ITGB1-KO and Src inhibition (Supplemental Figure 9B and Figure 7, C and D). ITGB1-KO itself had no proliferation disadvantage in SCLC cells (Supplemental Figure 9C) but hindered migration and invasion upon fibronectin and ANG-2–dependent stimulation (Supplemental Figure 9, D and E, and Supplemental Figure 10).

In a next step, we investigated the functional relevance of ADAM9, as it is a target downstream of ANG-2. ADAM9 knockdown was able to stop ANG-2–mediated SCLC migration (Supplemental Figure 11, A–C). In addition, ADAM9 correlated with ITGB1 expression in human SCLC samples and cell lines (Supplemental Figure 11, D and E).

**Figure 8:**
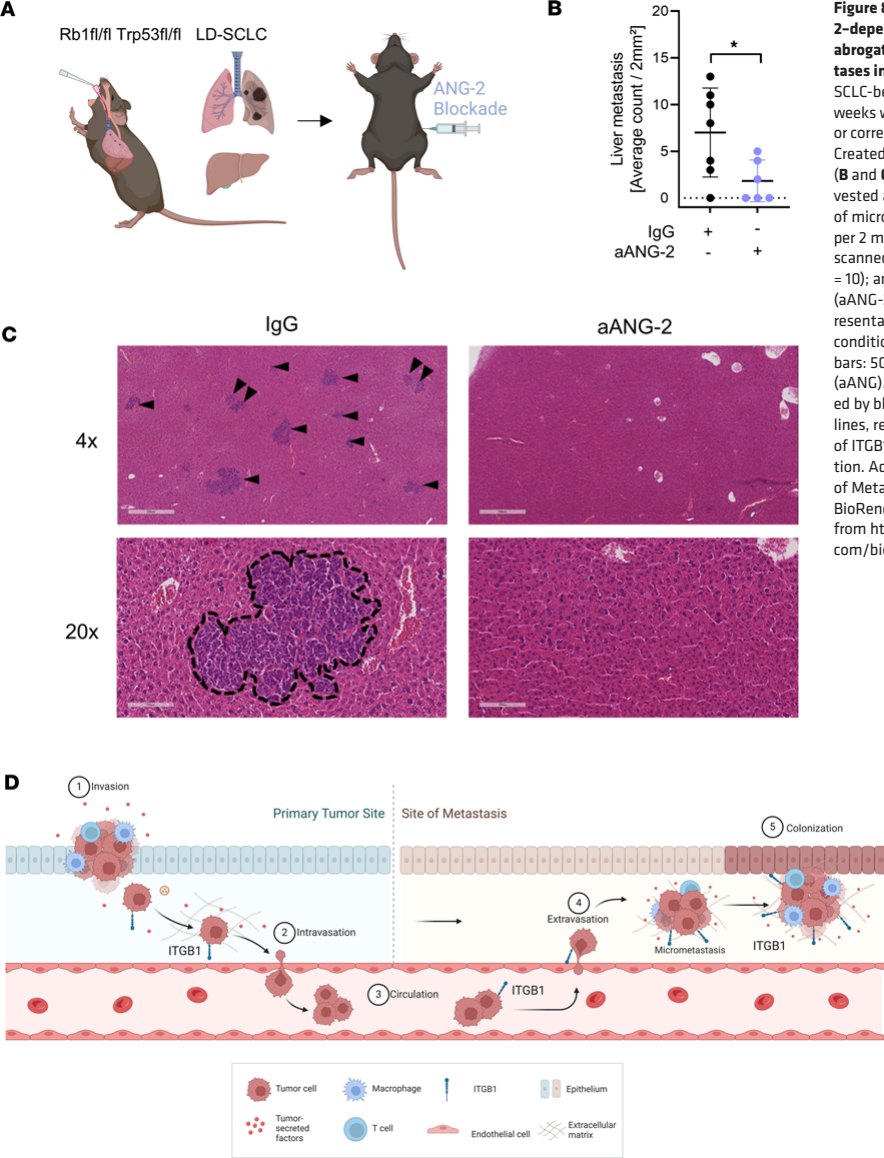
Blockade of ANG-2–dependent ITGB1 signaling abrogates SCLC liver metastases in vivo. (**A**) Scheme of SCLC-bearing mice treated for 2 weeks with anti–ANG-2 antibody or corresponding IgG control. Created with BioRender.com. (**B** and **C**) Liver tissue was harvested and the average number of microscopic liver metastases per 2 mm^2^ was counted by scanned H&E slides. IgG (black; *n* = 10); anti–ANG-2 monotherapy (aANG-2, light blue; *n* = 6). Representative images for relevant conditions are shown. Scale bars: 500 μm (IgG) and 100 μm (aANG). Metastases are indicated by black arrows and dashed lines, respectively. (**D**) The role of ITGB1 in intra- and extravasation. Adapted from “Overview of Metastatic Cascade,” by BioRender.com (2022). Retrieved from https://app.biorender.com/biorender-templates

We further investigated ANG-2 blockade in vivo as a potential treatment strategy to inhibit SCLC metastasis. Mice with limited stage of disease (LD) have been treated with either anti–ANG-2 and IgG control (Figure 8A). Analyzing H&E stains of liver tissue indicated that ANG-2 blockade abrogated SCLC liver metastasis, whereby 50% of anti–ANG-2–treated mice did not show any liver metastasis (Figure 8, B and C). In serum samples of treated mice, we were able to identify the immunomodulatory effect assisting the antimetastatic effect of anti–ANG-2 (Supplemental Figure 12). Macrophages in liver metastasis of untreated mice showed increased expression of PD-1 and CTLA-4 that were reduced upon anti–ANG-2 treatment (Supplemental Figure 12, A and C). Both immune checkpoints are known to negatively regulate the tumor suppressor functions of macrophages in cancer (20, 21). Moreover, in vivo cytokine analysis revealed an antitumorigenic M1 signature upon anti–ANG-2 therapy (Supplemental Figure 12, B, D, and E). Taken together, these findings demonstrate that ITGB1 fulfils a pivotal role in the metastatic cascade of SCLC (Figure 8D) in an ANG-2–dependent manner.

### Targeting ANG-2/ITGB1 signaling combined with antiangiogenic/anti–PD-1 therapy synergistically prolongs survival.

We previously demonstrated synergistic treatment effects by combining antiangiogenic VEGFR signaling blockade with anti–PD-L1 therapy in mice with SCLC. However, we found an increased formation of tumor metastasis upon antiangiogenic treatment. The blockade of VEGF/VEGFR signaling has been described to drive EMT in different tumor entities (22–24). In line with these data, we found that VEGFR inhibition with Vatalanib (PTK787), as well as KO of tumor VEGF-A via CRISPR-Cas9, resulted in increased formation of liver and rib cage metastasis with strong Vimentin expression in mice with SCLC (Supplemental Figure 13).

**Figure 9:**
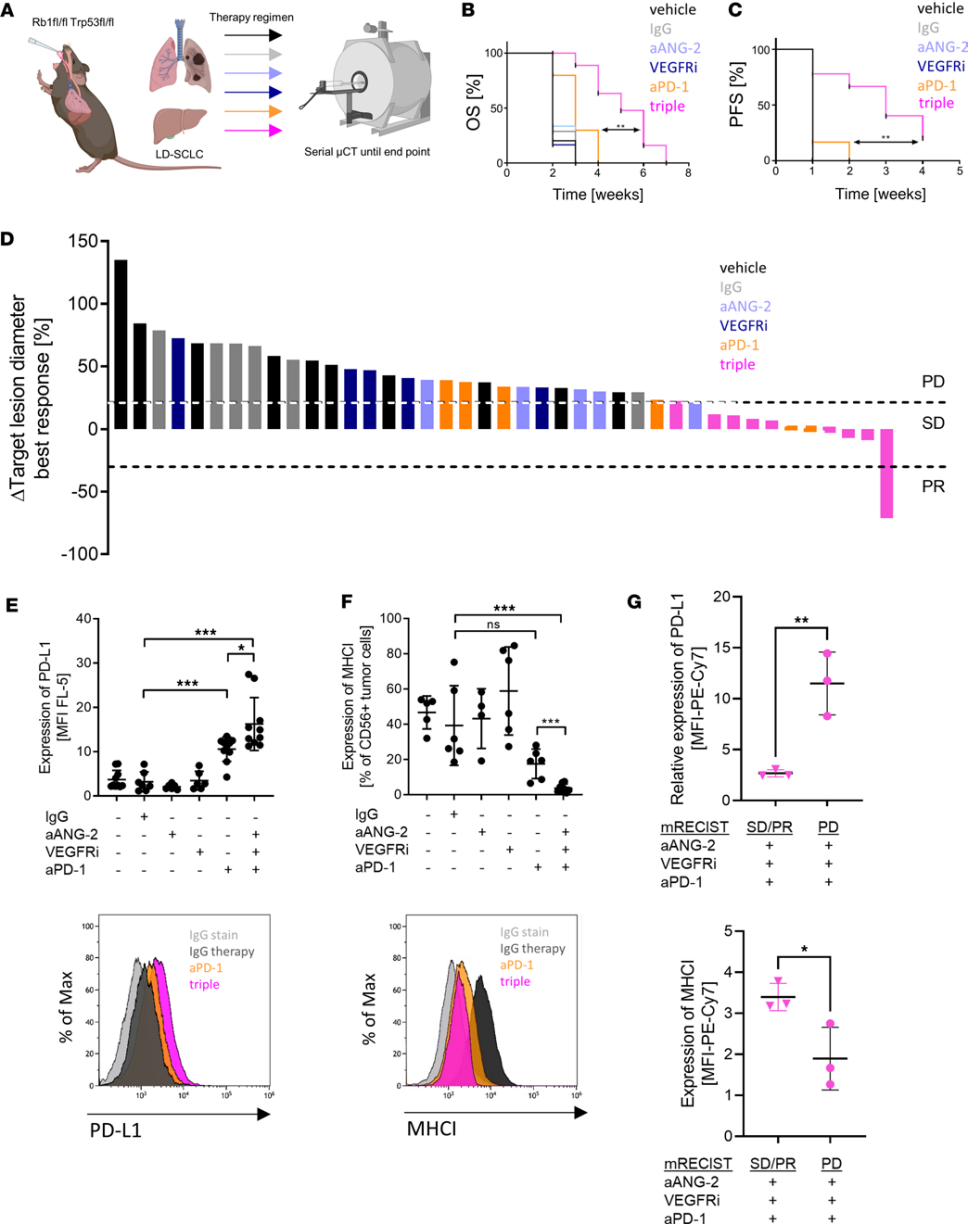
Synergistic treatment effects by combined blockade of ANG-2/ITGB1, VEGFR, and anti–PD-1 in mice with SCLC. (**A**) Schematic of SCLC-bearing mice treated with vehicle control (black; *n* = 10), IgG control (gray; *n* = 7), VEGFR inhibitor monotherapy (VEGFRi, dark blue; *n* = 6), anti–ANG-2 monotherapy (aANG-2, light blue; *n* = 6), anti–PD-1 monotherapy (aPD-1, orange; *n* = 10), and anti–ANG-2/VEGFR inhibitor/anti–PD-1 triple combination therapy (triple, pink; *n* = 10). Created with BioRender.com. (**B** and **C**) Overall survival (OS) and progression-free survival (PFS) were determined based on mouse adapted RECIST v1.1 criteria determined by serial μCT of mice from the 6 therapy cohorts. Statistical analysis was done using Mantel-Cox test (**P < 0.01). (**D**) Change in target lesion diameter from the start of therapy upon best response to treatment. The received therapy is indicated by the color code. PD, progressive disease; SD, stable disease; PR, partial response. (**E** and **F**) Tumor cells isolated from primary SCLC tumors under therapy were analyzed for PD-L1 and MHC class I expression by flow cytometry. (**G**) Tumor cells isolated from primary SCLC tumors under therapy were analyzed for PD-L1 and MHC class I expression under response and resistance by flow cytometry. Statistical analysis was done using the 2-tailed Student’s *t* test (*P < 0.05; **P < 0.01; ***P < 0.001; data are shown as mean ± SEM).

Thus, we here aimed to unravel whether ANG-2 blockade could abrogate this VEGFR blockade–induced metastatic phenotype in vivo. For this purpose, we applied an inhibitor of ANG-2/VEGF-A, BI836880. It works bispecifically in humans, but in mice, BI836880 selectively blocks ANG-2 alone (11). Strikingly, ANG-2–targeted treatment inhibited the formation of liver metastases upon VEGFR inhibition. As recent clinical data revealed that metastases are an independent prognostic factor for overall survival (OS) in patients with SCLC treated with immune checkpoint blockade (25), we next evaluated whether ANG-2 blockade improves therapeutic outcome in SCLC. We applied ANG-2–targeted treatment in combination with a monoclonal anti–PD-1 targeting antibody and VEGFR inhibitor (Vatalanib) in autochthonous SCLC mice (Figure 9A). By using Vatalanib in combination, we ensure corresponding blocking effects similarly occurring in patients treated with BI836880, since this compound acts bispecifically against ANG-2 and VEGF-A in humans.

To determine synergistic treatment effects using a statistical factor model, we also tested the corresponding monotherapies in vivo. Interestingly, combined ANG-2/VEGFR/PD-1 triple blockade significantly prolonged OS (Figure 9B) and progression-free survival (PFS) (Figure 9C) compared with anti–PD-1 monotherapy and the other therapies investigated (Supplemental Figure 14, A and B, and Supplemental Tables 5 and 6). Considering target lesion diameters at the time point of best response to therapy, verified by μCT, 8 of 9 mice (88.89%) treated with ANG-2/VEGFR/PD-1 triple blockade presented tumors with stable disease (SD) or partial response (PR). Under anti–PD-1 monotherapy, only 2 of 6 mice (33.34%) showed SD tumors (Figure 9D).

For synergy analysis, we used a statistical factor model (R survival package), taking the hazard ratio of each monotherapy into account. We validated the suitability of the proportional hazard model for the factor-based analysis and revealed similar results in the survival analysis as the Mantel-Cox test (Supplemental Figure 14, C and D). Welch’s correction and the proportional hazard ratio model (Supplemental Tables 7 and 8) revealed significant differences between the expected and the observed OS and PFS upon blockade of ANG-2, VEGFR, and PD-1 after sufficient randomization of the treated mice, indicating synergistic therapeutic effects (Supplemental Figure 14, F and G).

We further analyzed SCLC primary tumor cell ex vivo by flow cytometry in order to investigate mechanisms of therapy resistance. We found that SCLC tumor cells highly expressed PD-L1 upon resistance to anti–PD-1 (Figure 9E). In addition, we detected MHC-I loss on SCLC tumor cells growing out under anti–PD-1 and upon combined blockade of ANG-2, VEGFR, and PD-1 (Figure 9F). Both increased PD-L1 and decreased MHC-I are indicators of resistance and have been truly acquired upon therapy resistance comparing responder mice and progressor mice treated with combined blockade of ANG-2, VEGFR, and PD-1 (Figure 9G).

### SCLC liver metastases resemble the primary tumor regarding T cell infiltration.

**Figure 10:**
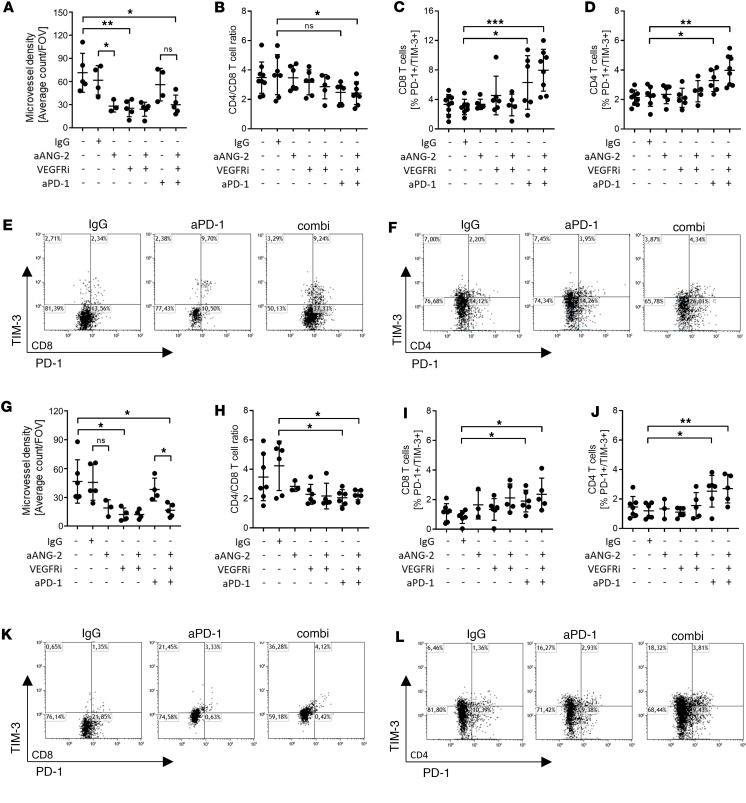
Resistance to anti–PD-1 therapy–induced exhausted T cells in SCLC primary and liver metastases. SCLC-bearing mice were analyzed for microvessel density and T cell infiltrates based on IHC and flow cytometry upon detection of progressive disease based on mouse adapted RECIST v1.1. (**A**–**F**) Corresponding to primary SCLC samples treated as follows: vehicle control (vehicle; *n* = 9), IgG control (IgG; *n* = 7), VEGFR inhibitor monotherapy (VEGFRi; *n* = 6), anti–ANG-2 monotherapy (aANG-2; *n* = 6), anti–ANG-2 and VEGFR inhibitor combination therapy (aANG-2/VEGFRi; *n* = 5), anti–PD-1 monotherapy (aPD-1; *n* = 6), and anti–ANG-2/VEGFR inhibitor/anti–PD-1 triple combination therapy (triple; *n* = 8). (**G**–**L**) Corresponding to SCLC liver metastases: vehicle (*n* = 7), IgG control (IgG; *n* = 6), VEGFR inhibitor monotherapy (VEGFRi; *n* = 6), anti–ANG-2 monotherapy (aANG-2; *n* = 3), anti–ANG-2 and VEGFR inhibitor combination therapy (aANG-2/VEGFRi; *n* = 5), anti–PD-1 monotherapy (aPD-1; *n* = 6), and anti–ANG-2/VEGFR inhibitor/anti–PD-1 triple combination therapy (triple; *n* = 5). (**A** and **G**) Microvessel density was determined based on IHC using ImageJ to measure CD31^+^ counts in a field of view (FOV) of 200 μm^2^. Statistical analysis was done using the 2-tailed Student’s *t* test (*P < 0.05; **P < 0.01; ***P < 0.001; data are shown as mean ± SEM). (**B** and **H**) The ratio of CD4^+^ T cells versus CD8^+^ T cells is compared in the different therapy cohorts based on flow cytometry. (**C** and **I**) The fraction of PD-1 and TIM-3 double-positive CD8^+^ T cells is determined for each therapy cohort based on flow cytometry. (**D** and **J**) The fraction of PD-1 and TIM-3 double-positive CD4^+^ T cells is determined for each therapy cohort based on flow cytometry. (**E** and **K**) Dot plots of flow cytometry of CD8^+^ T cells of 1 representative experiment are shown. (**F** and **L**) Dot plots of flow cytometry of CD4^+^ T cells of 1 representative experiment are shown. Statistical analysis was done using the 2-tailed Student’s *t* test (*P < 0.05; **P < 0.01; ***P < 0.001; data are shown as mean ± SEM). Representative IHC stains of primary SCLC and SCLC liver metastases are shown in Supplemental Figures 15–18.

Since increased T cell infiltration in tumor tissue is associated with efficacy to immune checkpoint blockade (26), we analyzed SCLC tissues obtained from lungs and livers by IHC (Supplemental Figures 15–18). Murine SCLCs resembled the human SCLCs in regard to neuroendocrine marker expression determined by NCAM (Supplemental Figures 15 and 17), proliferative index determined by KI-67 (Supplemental Figures 15 and 17, and Supplemental Figure 19D), and vascular density determined by CD31 (Figure 10, A and G, and Supplemental Figures 16 and 18). Thereby, antiangiogenic treatment decreased vessel density in primary and metastases alike. Moreover, combined blockade of VEGFR, ANG-2 and PD-1 in a triple blockade improved CD8^+^ T cell infiltration in both, primary lung tumor, and liver metastasis (Figure 10, B and H, and Supplemental Figures 16 and 18). However, tumors in lung and liver progressed under ongoing therapy presented with an exhausted T cell phenotype indicated by simultaneous PD-1 and TIM-3 expression (Figure 10, C–F and I–L, and Supplemental Figure 19, B and C).

Taken together, combining blockade of ANG-2, VEGFR, and PD-1 synergistically prolonged OS and PFS of SCLC-bearing mice and was accompanied by better CD8^+^ T cell infiltration into tumor tissue in lung and liver. However, primary SCLC tumors acquire immune checkpoint blockade resistance likely by PD-L1 expression and MHC-I loss accompanying the end of SCLC therapy in mice.

## Discussion

In this study, we identify a therapeutically tractable prometastatic signaling pathway mediated via ITGB1-FAK/SRC signaling, driving the invasive nature of SCLC. Previous studies have linked integrins to poor prognosis in SCLC, but the underlying mechanism remained elusive for more than 15 years (27). Mechanistically, we show that ITGB1 signaling in SCLC tumor cells induced an EMT-like phenotype via FAK/SRC signaling and that ITGB1 fulfilled essential roles in intra- and extravasation of SCLC cells. We further demonstrate that blocking ANG-2/ITGB1 signaling suppressed tumor cell invasion and the formation of metastasis in an autochthonous SCLC mouse model. In line with these findings, previous studies have shown that ANG-2/ITGB1–dependent signaling induces EMT and invasion, and thereby contributes to metastasis formation in non-SCLC (NSCLC) (19) and gliomas (18). Our preclinical data on this function of ITGB1 are strengthened by clinical data from a collection of matched primary tumor and liver metastasis samples from patients with SCLC that indicate a clear/significant increase of ITGB1 in liver metastasis in comparison with the primary tumor.

Our data are in line with previous findings distinguishing a key role of integrins, such as ITGB1, for tumor metastasis in breast cancer (28). Moreover, Gengenbacher and colleagues recently discovered that ANG-2/TIE-2 signaling is essential for the metastatic process in intratumoral lymphatics involving pericytes in an autochthonous melanoma model and an orthotopic mammary carcinoma model (29).

Regarding the clinical application, our data strongly indicate that tumor cell–dependent ANG-2/ITGB1–mediated invasion and metastasis is a highly promising therapeutic target to prevent the formation of liver metastasis in patients with SCLC. Recent clinical data highlight the relevance of metastasis for predictive outcome of patients with SCLC treated with immune checkpoint inhibition in combination with chemotherapy (30, 31). Thus, an effective targeted treatment approach that suppresses tumor cell invasion and metastasis in patients with SCLC is of high clinical relevance. We and others show that combining immunomodulatory therapies, such as anti-VEGFR with immune checkpoint inhibition, improves long-term treatment outcome (NCT02734004; NCT03417895; NCT04055792; NCT04453930) (32, 33). We here demonstrate that the addition of ANG-2–targeted therapy suppresses the formation of metastasis and displays synergistic treatment effects in combination with anti–PD-1 blockade and VEGFR inhibition. This finding is line with recent publications focusing on NSCLC (11, 34). Martinez-Usatorre et al. applied a combined triple blockade targeting VEGF-A by B20 antibody, ANG-2 BI-836880 and PD-1 in an autochthonous NSCLC mouse model driven by mutated *KRAS* and *TP53*. They detected an improved infiltration of T cells into the tumor mass upon anti–ANG-2 treatment. However, they did not show increased efficacy upon adding anti–PD-1 in NSCLC (34). In a Lewis lung 2 (LL/2) syngeneic lung cancer mouse model, where a combined triple blockade targeting VEGFR by Vatalanib, ANG-2 by BI-836880, and PD-1 by RMP1-14 was used, the authors have shown improved in vivo antitumor efficacy (11). Moreover, they explored extensive binding studies and pharmacodynamics of BI-836880. They postulated that BI-836880 inhibited endothelial cell proliferation and survival and led to decreased vessel density in vivo and, thereby, to improved CD8^+^ T cell infiltration, which is in line with our data (11). These diverging therapy outcomes in NSCLC may be caused by a different composition of the tumor microenvironment regarding macrophages and the different compound used to block VEGF/VEGFR signaling. In one of our previous studies using the autochthonous SCLC mouse model, we have shown that anti–VEGF-A directly affects T cell exhaustion independently of major vascular effects and, thereby, contributes to prolonged survival upon anti–PD-L1 (26). With our current study, we can further add that SCLC metastases mimicked the primary tumor regarding decreased intratumoral vessel density, T cell infiltration, and exhaustion when using a small-molecule inhibitor for VEGFR.

Of note, it has been described that ANG-2 blockade modulated monocytes and macrophages in neuroinflammatory syndromes (35). The authors studied the ANG-2 antagonistic role regarding TIE-2 signaling in a demyelinating CNS autoinflammatory disease. Interestingly, ANG-2 blockade was accompanied by inhibition of α5β1 integrin activation in microglia (35). These findings together with our findings suggest that ANG-2 blockade is able to modulate TIE-2–dependent and ITGB1-dependent signaling simultaneously. We have here shown that TIE-2 seems to play a minor role in SCLC progression. Thus, the results from Li et al. (35) combined with our study suggest that the ANG-2–mediated ITGB1-dependent mechanism likely dominates in the tumor progression setting and the ANG-2–mediated TIE-2–dependent mechanism in an autoimmune setting.

Taken together, ANG-2/ITGB1 signaling promotes tumor metastasis in SCLC that is therapeutically vulnerable. Thus, our data strongly indicate that patients with SCLC may benefit from ANG-2–targeted treatment in combination with established therapy regimens.

## Methods

### Sex as a biological variable.

In animal experiments, male and female mice with a minimal weight of 20 g and a minimal age of 6 weeks were included. Regarding the analysis of human data, patients have not been excluded from the evaluation based on their sex. In matched patient samples, by chance, only 1 female patient was included. Sex is annotated in Supplemental Table 2.

### Animal experiments.

The genetically engineered mouse model of SCLC is driven by a Cre-inducible conditional *Rb1* and *Tp53* KO, as previously described (16). Male and female C57BL/6J mice with a minimal weight of 20 g and a minimal age of 6 weeks were anesthetized with Ketamin 100 mg/kg/body weight [BW] i.p.)/Xylazin (0.5 mg/kg/BW i.p.), and Adeno-Cre was applied intratracheally. Viral vectors were provided by the University of Iowa Viral Vector Core (http://www.medicine.uiowa.edu/vectorcore) Iowa City, Iowa, USA. Similar findings are reported for both sexes. Serial μCT was performed to monitor tumor induction, response to therapy and progression under therapy. Mice were randomized to the 6 therapy cohorts before tumor induction. The target lesion diameters at the start of treatment were similarly distributed between the groups.

Twenty weeks after virus inhalation, once a week, mice were anesthetized using isoflurane and receive a serial μCT scan (LaTheta mCT, Hitachi Alcoa Medical Ltd.). Upon a measurable target lesion ≥ 1 mm in diameter, therapy regimens were started. SCLC cohorts comprised 6 therapy groups, and all therapies were given every 3 days simultaneously: (Group 1) vehicle (PBS); (Group 2) IgG (corresponding monotherapy IgGs [Southern Biotech] diluted in PBS); (Group 3) anti–ANG-2 (BI836880, Lot no. 314A80003, bispecific nanobody consisting of 1 domain binding human VEGF-A, 1 domain binding both human and murine ANG-2, and both domains, provided by Boehringer Ingelheim; ref. 11) with a concentration of 15 mg/kg/BW i.p. per injection; (Group 4) the VEGFR–tyrosine kinase inhibitor (TKI) Vatalanib (PTK787, Med Chem Express, lot no. 25923, catalog HY-12018/CS-0149, diluted in water) with a concentration of 75 mg/kg/BW per oral gavage; (Group 5) anti–PD-1 (monoclonal antibody RMP1-14, lot no. 40517M2, catalog BE0146, provided by Boehringer Ingelheim) with a concentration of 10 mg/kg/BW i.p. per injection; and (Group 6) combined triple combination comprising anti–ANG-2 (15 mg/kg/BW i.p.), Vatalanib (75 mg/kg/BW p.o.), and anti- PD-1 (10 mg/kg/BW i.p.) applied simultaneously.

Tumor response and progress under ongoing treatment was accessed by mouse-adapted RECIST criteria v1.1, as published previously (26). Slice thickness was adapted to 0.3 mm. The first dose was given upon target lesion identification and baseline evaluation, maximally 1 day before. Response criteria to evaluate the target lesion were maintained regarding diameter fold change. Complete response (CR) referred to a decrease of 100%, PR was indicated upon a > 30% reduction, progressive disease (PD) referred to an increase of > 20% and/or new lung lesions, and SD was termed upon a diameter change that did not qualify for PR or PD. μCT data were analyzed using OsiriX-DICOM viewer (aycan Digitalsysteme GmbH).

OS and PFS achieved with the different therapy regimens were analyzed as Kaplan-Meier curves and statistically evaluated using Mantel-Cox Prism (GraphPad, V8.0) and the proportional hazard model (R survival package, R Core Team, 3.6.3). Synergy analysis of the triple combination with anti–ANG-2, VEGFR inhibitor, and anti–PD-1 was performed with the proportional hazard model. Ratios and *P* values are included in Supplemental Tables 5–8. Scripts are available upon request. To study intra- and extravasation processes of SCLC cells, different routes of tumor cell injection were carried out in immunocompetent male and female C57BL/6J mice with a minimal weight of 20 g. For intravasation measures, mice were anesthetized with Ketamin (100 mg/kg/BW i.p.)/Xylazin (0.5 mg/kg/BW i.p.) and warmed. 5 million tumor cells were injected into the right thorax. The scapula served as an orientation point leading to the fourth or fifth intercostal space. The tumor growth was monitored by serial μCT scans (LaTheta mCT, Hitachi Alcoa Medical Ltd.) under isoflurane anesthesia. For extravasation measures, mice were mechanically restrained and warmed. 1 million tumor cells were injected in the tail vein. The tumor growth and metastasis were monitored by serial MRI scans using a 3.0T MRI (Philips) with a dedicated small animal coil (diameter 40 mm; Philips Research) under isoflurane anesthesia.

### IHC.

Murine organs were harvested, and paraffin embedded. Tissue sections (3 μm) were deparaffinized, and IHC was performed using the LabVision Autostainer-480S (Thermo Fisher Scientific). Staining was done using H&E, primary antibodies, and the Secondary-Histofine-Simple-Stain (SHSS) antibody detection kit (Medac). Slides were scanned by the Panoramic-250 slide scanner (3D Histech).

Human primary SCLC and metastasis were diagnosed based on histological examination by trained lung pathologists. Clinicopathologic characteristics are listed in Supplemental Table 2. Pictures were acquired with a Leica-DM-5500 B Microscope. Primary antibodies against ITGB1 (Santa Cruz, 1:100, A4) and Vimentin (Thermo Fisher Scientific, 1:200, SP20) were used for characterization of matched metastases in patients. Primary antibodies (anti-murine): KI-67 (Cell Marque, 1:50, SP6), CD31 (Dianova, 1:25, SZ31), CD56 (NCAM) (Abcam, 1:50, polyclonal, ab95153), CD4 (Abcam, 1:1,000, EPR19514), CD8 (Abcam, 1:200, polyclonal, ab203035), E-cadherin (Cell Signaling Technology, 1:100, 24E10), Snail (Abcam, 1:200, polyclonal, ab53519), ANG-2 (1:50, polyclonal, ABIN7254971), ITGB1 (Cell Signaling Technology, 1:100, D6S1W), and Vimentin (Thermo Fisher Scientific, 1:50, SP20). Secondary antibodies were purchased from ImmunoLogic (BrightVision+), and staining was performed using the LabVision Autostainer 480S (Thermo Fisher Scientific).

### Flow cytometry.

Organs of mice were harvested, and cells were isolated by mechanical dissociation using 40 μm cell strainers (BD Falcon). RBCs were lysed by ACK lysis buffer (Life Technologies), and cells were washed with PBS. Purified primary tumor cells, macrophages, and T cells were stained for 30 minutes at 4°C for flow cytometry using primary antibodies and isotype controls, both obtained from BioLegend if not otherwise specified. Primary antibodies (anti-murine): ITGB1 (Pacific Blue, HMβ1-1), CD3 (Alexa Fluor 700, 17A2), CD4 (PE-Dazzle594, GK1.5), CD45 (APC-Cy7, 30-F11), CD49e (PE-Cy7, 5H10-27), CTLA-4 (PE, UC10-4B9), CD56 (R&D Systems, APC, 809220), CD61 (PerCP-Cy5.5, 2C9.G2), CD8a (FITC, Pacific Blue, 53-6.7), H-2Kb (Pacific Blue, AF6-88.5), PD-1 (APC, 29F.1A12), PD-L1 (PE-Cy7, 10F.9G2), TIE-2 (PE, TEK4), TIM-3 (PerCP-Cy5.5, B8.2C12), rat IgG2aΚ (FITC, PE, PerCP-Cy5.5, APC, Alexa Fluor 700), PE-Dazzle594 Armenian hamster IgG (PE-Dazzle594), rat IgG2bΚ (PE-Cy7), and mouse BALB/c IgG2aΚ (Pacific Blue). In addition, the Zombie Aqua Fixable Viability Kit (BioLegend) was used. Flow cytometry was performed on a Gallios 10/3 (Beckman Coulter), and data were analyzed using FlowJo (Tree Star v7.6.1).

### Cytokine array.

Briefly, whole blood was incubated for 1 hour at room temperature (RT) in 500Z-Gel Microvettes (Sarstedt) and then centrifuged for 5 minutes at 20°C with 10,000*g*. In total, 75 μL of harvested serum were analyzed in duplicates using the Mouse Cytokine/Chemokine 31-Plex Discovery Assay Array (MilliporeSigma), according to the manufacturer’s protocol, and measured using the Luminex 100 system by Eve Technologies Corporation. The biomarkers included to following: Eotaxin, G-CSF, GM-CSF, IFN-γ, IL-1α, IL-1β, IL-2, IL-3, IL-4, IL-5, IL-6, IL-7, IL-9, IL-10, IL-12p40, IL-12p70, IL-13, IL-15, IL-17A, IP-10, KC, LIF, LIX, MCP-1, M-CSF, MIG, MIP-1α, MIP-1β, MIP-2, RANTES, TNF-α, and VEGF-A. *Z* scores of log_10_ of raw biomarker levels were calculated and a heatmap with Morpheus (Broad Institute) was generated using hierarchical clustering with metric of 1-Pearson correlation.

### Cell culture.

Human lung cancer cell lines were provided by Roman K. Thomas (Department of Translational Genomics, University of Cologne) and Reinhard Büttner (Institute for Pathology, University Hospital Cologne, Germany). Murine SCLC cells were isolated and prepared as single cells, from harvested lungs as primary tumor cells and from livers as metastasized tumor cells, of mice harboring extensive SCLC using 40 μm cell strainer (VWR) and ACK lysis buffer.

SCLC cells were cultured in RPMI medium (Life Technologies) with 10% FCS (MilliporeSigma) and 1% antibiotic (penicillin + streptomycin, Life Technologies) and checked for mycoplasma contamination by PCR on a regular basis.

The murine cell lines are classified as stable cell lines after 5 passages and are usable in functional assays. For stimulation experiments, cells were seeded and stimulated using 20 ng/mL murine VEGF165 (PeproTech), also known as VEGF-A, and 2.5 μg/mL Vatalanib, 100 nM Saracatinib, 250 ng/mL ANG-2 (R&D Systems), and Fibronectin (Stemcell Technologies) with 1 μg/cm^2^ and 10 μg/cm^2^ according to manufacturer’s advices. Assays and time intervals are analyzed as indicated.

### KO and knockdown generation.

KO of tumor VEGF-A and ITGB1 in mouse-derived SCLC cell clones was performed by RNA-guided Cas9 using Santa Cruz CRISPR constructs according to manufacturer’s advices. Cells were seeded 1 day before transfection on 6 wells and were transfected using Lipofectamine LTX Plus reagent according to the manufacturer’s protocol. Three days after transfection, SCLC cells were sorted as single cells into 96-well plates, 1 cell per well in collaboration with the Institute of Virology, University Hospital Cologne. Individual clonal cell lines were generated and analyzed by Western blot and flow cytometry for sufficient KO of VEGF-A and ITGB1. ADAM9 knockdowns had been generated based on siRNA (sc-41409) provided by Santa Cruz according manufacturer’s advices.

### Migration assay.

Twelve-well plates were coated with Fibronectin for 60 minutes in 37°C incubator. Afterward, each well was washed with PBS. In total, 70,000 murine SCLC cells were seeded and grown until a confluency of 70%–80% before applying a scratch to the bottom of the well. Detached cells were removed by washing with PBS. Fresh medium was applied, and scratches were analyzed after 48 hours by microscope. Wound healing was analyzed using ImageJ software (NIH). The same procedure applied to other stimulations carried out in migration assays.

### Invasion assay.

The Boyden Chamber system (Greiner Bio One GMBH) was set into a 24-well plate filled with medium containing the respective chemoattractant. For the invasion assay, 30,000 cells were placed into the chamber, which is separated from the medium by a porous membrane of 0.4 μm pore size. The upper chamber was filled serum-free medium. The lower compartment was supplemented with RPMI medium 1640 containing 10% FBS. The cells were incubated for 48 hours. Cells in the interior part of the insert were removed with a cotton swab. Cells on the bottom of the membrane were fixed with 4% paraformaldehyde for 20 minutes at RT and stained with 1% crystal violet solution for 5 minutes. The excess dye was washed away and the chamber was dried for 20 minutes at RT. Stained cells were analyzed by microscope and 5 representative 4× images were taken from the entire surface. The threshold method of ImageJ software was applied to analyze the crytstal violet–labeled density/area covered by tumor cells.

### Western blot.

Cells were digested with lysis buffer, and protein concentrations were determined by a BCA Protein Assay (Pierce). For SDS-PAGE, cell lysates were mixed with 4× NuPage LDS buffer and sample-reducing agent (10×) (Life Technologies), incubated at 80°C for 10 minutes, and loaded on NuPAGE Bis-Tris Gels 4%–12% (Invitrogen) run 130V for approximately 90 minutes. The transfer of the separated proteins on a nitrocellulose Membrane (Amersham Hybond-C Extra) was performed by wet-blotting in a XCell II Blot Module (Life Technologies). The membrane was blocked with Blocking Buffer (4% milk in TBST) and incubated overnight with the primary antibodies diluted in Blocking Buffer. Primary antibodies included the following: ADAM9 (Cell Signaling Technology, 1:1,000, D64B5), E-cadherin (Cell Signaling Technology, 1:1,000, 24E10), Snail (Cell Signaling Technology, 1:1,000, C15D3), Vimentin (Abcam, 1:500, EPR3776), p–Fak Tyr397 (Cell Signaling Technology, 1:1,000, polyclonal, 3283), FAK (Cell Signaling Technology, 1:1,000, D2R2E), p–SRC Tyr416 (Cell Signaling Technology, 1:500, polyclonal, 2101), SRC (Cell Signaling Technology, 1:1,000, 36D10), and β-actin (MP Biomedicals, 1:3,000, C4).

After washing for 30 minutes with TBST, the membrane was incubated with secondary HRP-conjugated antibody 1:3,000 in TBST for 1 hour at RT (anti–rabbit-HRP and anti–mouse-HRP antibodies; both from MilliporeSigma) and developed on ECL Hyperfilm with ECL Western blot detection System (GE Healthcare). The RTK assay (R&D Systems) was performed according to manufacturer’s instructions using 300 μg protein.

### Gene expression analysis.

For analysis of patients with SCLC, RNA-Seq and clinical annotation were obtained from a published study (12). Association between ITGB1 expression and UICC tumor stage were tested using the nonparametric Jonckheere-Terpstra test. Single-sample Gene Set Enrichment Analysis (ssGSEA) was performed using the “GSVA” R-package against the MSigDB Hallmark EMT gene set from which ITGB1 was removed. mRNA expression data from human SCLC cell lines from Polley and colleagues is provided by CellMinerCDB within the SCLC NCI/DTP data set (15, 36). CellMinerCDB is a development of the Genomics and Pharmacology Facility, Developmental Therapeutics Branch (DTB), Center for Cancer Research (CCR), National Cancer Institute (NCI) prepared in collaboration with the cBio Center at the Dana-Farber Cancer Institute. The SCLC-CellMiner was accessed via https://discover.nci.nih.gov/SclcCellMinerCDB/ Heatmaps were generated by the provided heatmap tool, by setting the number of high/low response lines to display to 100, and by selecting row color scale. Expression of each of the genes provided in the gene sets “migration”, “apoptosis”, “mesenchymal”, and “epithelial” were correlated to ITGB1 expression across 64 SCLC cell lines (14). Pearson correlation coefficients per gene set were plotted as box plots, and a 1-sample Wilcoxon-test was used to determine whether the distribution of correlation coefficients per gene set was different from 0 (Supplemental Table 1).

### Statistics.

Statistics were done using Prism (GraphPad, V8.0), R survival package (R Core Team, 3.6.3), and SPSS (IBM, V24.0). Data are shown as mean ± SEM. *P* < 0.05 was regarded as significant. The used statistical tests such as ANOVA and Student’s *t* test are indicated in the figure legends.

### Study approval.

All human subject research was performed in strict accordance with approved protocols by the local ethics committee of the University of Cologne and with the recognized ethical guidelines of the Declaration of Helsinki. Tumor tissue (reference no. 13-091) was obtained during routine clinical procedures from patients with lung cancer providing written informed consent. Animal studies were performed in accordance with FELASA recommendations and with approval of the local Ethics Committee of Animal experiments (84-02.04.2015.A199; 81-02.04.2020.A026; 81-02.04.2020.A219; 81-02.04.2020.A328).

### Data availability.

Sequencing data have been uploaded in the MINSEQE-compliant public database Gene Expression Omnibus (GEO; accession no. GSE262409). Any additional material can be accessed from the corresponding authors. Values for all data points in graphs are reported in the Supporting Data Values file.

## Author contributions

Conceptualization was contributed by LM and RTU. Methodology was contributed by LM, CIO, CJO, MK, JB, MSE, FD, SB, MO, FH, FK, MH, R. Büttner, HCR, and RTU. Investigation was contributed by LM, CIO, CJO, MK, JB, MSE, SD, AC, CS, MN, R. Blazquez, FD, AF, and MLE. Visualization was contributed by LM, CIO, CJO, MK, JB, and FD. Funding acquisition was contributed by LM, JB, MO, R. Büttner, HCR, and RTU. Project administration was contributed by LM and RTU. Supervision was contributed by LM and RTU. Writing of the original draft was contributed by LM, CIO, CJO, MK, JB, and RTU. Review and editing were contributed by LM, CIO, CJO, MK, JB, MSE, SD, AC, CS, MN, MO, R. Blazquez, FD, AF, ME, SB, MO, FH, FK, MH, R. Büttner, HCR, and RTU.

## Supplementary Material

Supplemental data

Unedited blot and gel images

Supplemental table 1

Supplemental table 2

Supplemental table 3

Supplemental table 4

Supplemental table 5

Supplemental table 6

Supplemental table 7

Supplemental table 8

Supporting data values

## Version 1 05/22/2024

Electronic publication
