# Supplementary material for: Blocking the angiopoietin-2–dependent integrin β-1 signaling axis abrogates small cell lung cancer invasion and metastasis
Source: JCI Insight. 2024 May 22;9(10):e166402. doi: 10.1172/jci.insight.166402 (PMC11141935; doi:10.1172/jci.insight.166402)
Supplement: Supplemental data [file jciinsight-9-166402-s103.pdf]

Title

Blocking the Angiopoietin-2-dependent Integrin beta-1 signaling axis abrogates small cell lung cancer invasion and metastasis.

List of Supplemental Figures

Figure S1. ITGB3, ITGA5 and ITGA1 positively correlate with tumor stage in SCLC.

Figure S2. ITGB1 expression significantly correlates with migration markers in human SCLC cell lines.

Figure S3. ITGB1 protein expression of human SCLC cell lines.

Figure S4. Vimentin expression in liver metastasis of SCLC patients.

Figure S5. TIE-2 receptor expression is not altered upon extensive stage of disease on SCLC cells in vivo.

Figure S6. ANG-2 is not significantly increased in SCLC liver metastasis.

Figure S7. ITGB1 signaling is needed for extravasation of SCLC cells.

Figure S8. ANG-2-dependent ITGB1 mediated SCLC cell migration is regulated by SRC.

Figure S9. ITGB1 expression correlates with SCLC migration capacity and ANG-2 and Fibronectin provide similar transcripts associated to cell migration.

Figure S10. ITGB1 expression correlates invasion capacity.

Figure S11. ADAM9 is responsible for SCLC cell migration and regulated downstream of ITGB1.

Figure S12. Anti-Angiopoietin-2 therapy promotes an anti-tumorigenic macrophage phenotype in vivo.

Figure S13. Targeting Angiopoietin-2-dependent ITGB1 signaling counteracts a VEGF/VEGFR-induced metastatic SCLC phenotype.

Figure S14. Combining Angiopoietin-2-, VEGFR- and PD-1 blockade synergistically prolongs survival of SCLC bearing mice.

Figure S15. Murine primary SCLC tumors mimic the expression of NCAM and KI-67.

Figure S16. Murine primary SCLC tumors show increased T cell infiltration.

Figure S17. Murine SCLC liver metastases mimic the expression of NCAM and KI-67.

Figure S18. Murine SCLC liver metastases show increased T cell infiltration.

Figure S19. Liver metastases mimic primary SCLC regarding T cell exhaustion.

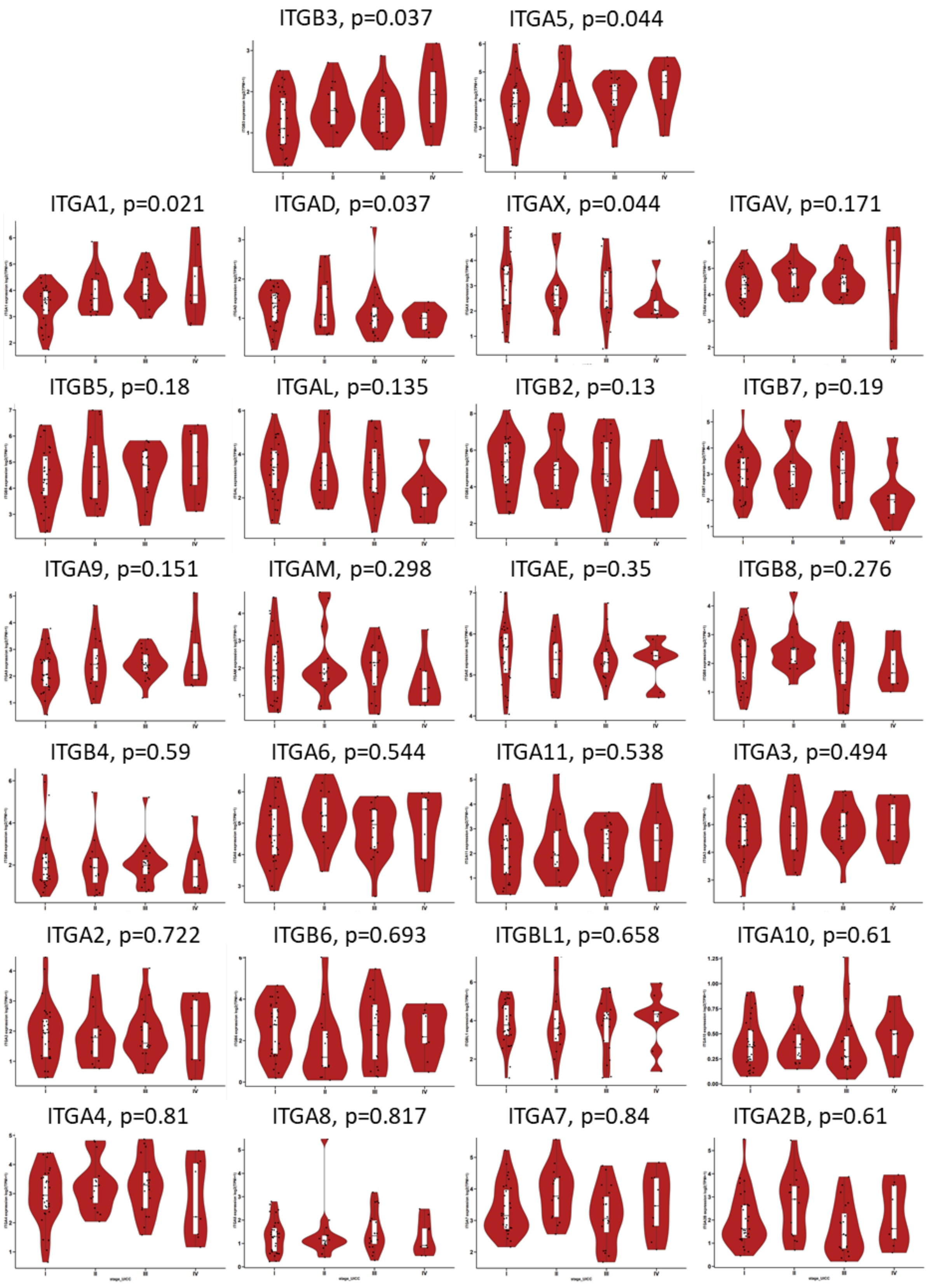

Figure S1. ITGB3, ITGA5 and ITGA1 positively correlate with tumor stage in SCLC. Human SCLC RNA-Seq data is analyzed regarding integrin correlations with tumor stage. Association between integrin gene expressions and UICC tumor stage is tested using the non-parametric Jonckheere-Terpstra test (n=75).

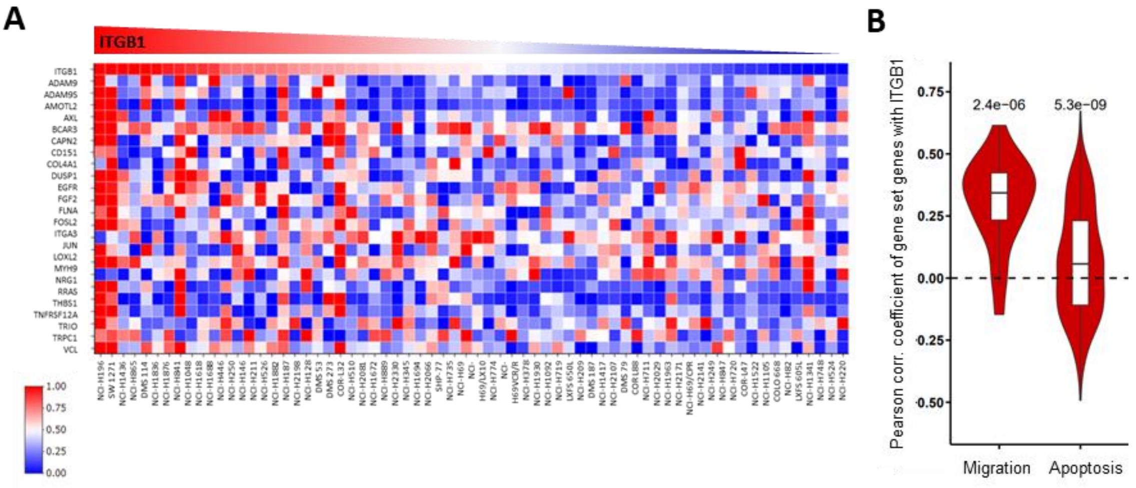

Figure S2. ITGB1 expression significantly correlates with migration markers in human SCLC cell lines.

(A) mRNA expression data for SCLC provide the "migration" and "apoptosis" gene sets in human SCLC cell lines (n=64). The heatmap is generated by the heatmap tool provided by SCLC-CellMiner. The correlation to ITGB1 expression is shown. (B) Pearson correlation coefficients with ITGB1 expression in human SCLC cell lines of all genes per "migration" and "apoptosis" gene set (p-values from one-sample Wilcoxon-tests for median corr. coefficient equal to zero per gene set; *, $p < 0.05$; **, $p < 0{,}01$; ***, $p < 0{,}001$). Data of mRNA gene expression and statistical analysis is listed in in the Supplemental Table S2.

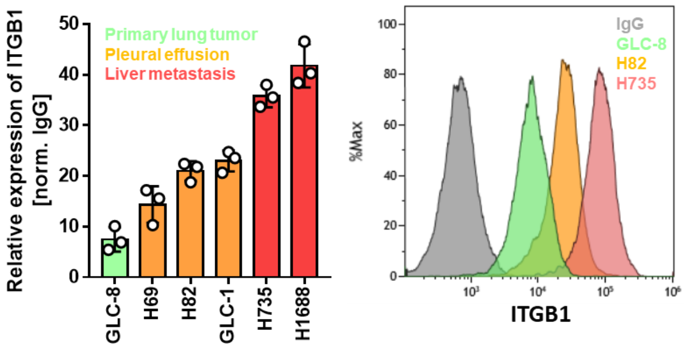

Figure S3. ITGB1 protein expression of human SCLC cell lines.

Surface expression of ITGB1 was analyzed in selected human SCLC cell lines from different tissues of origin by flow cytometry. Histogram indicates one representative experiment out of three. Error bars indicate SEM.

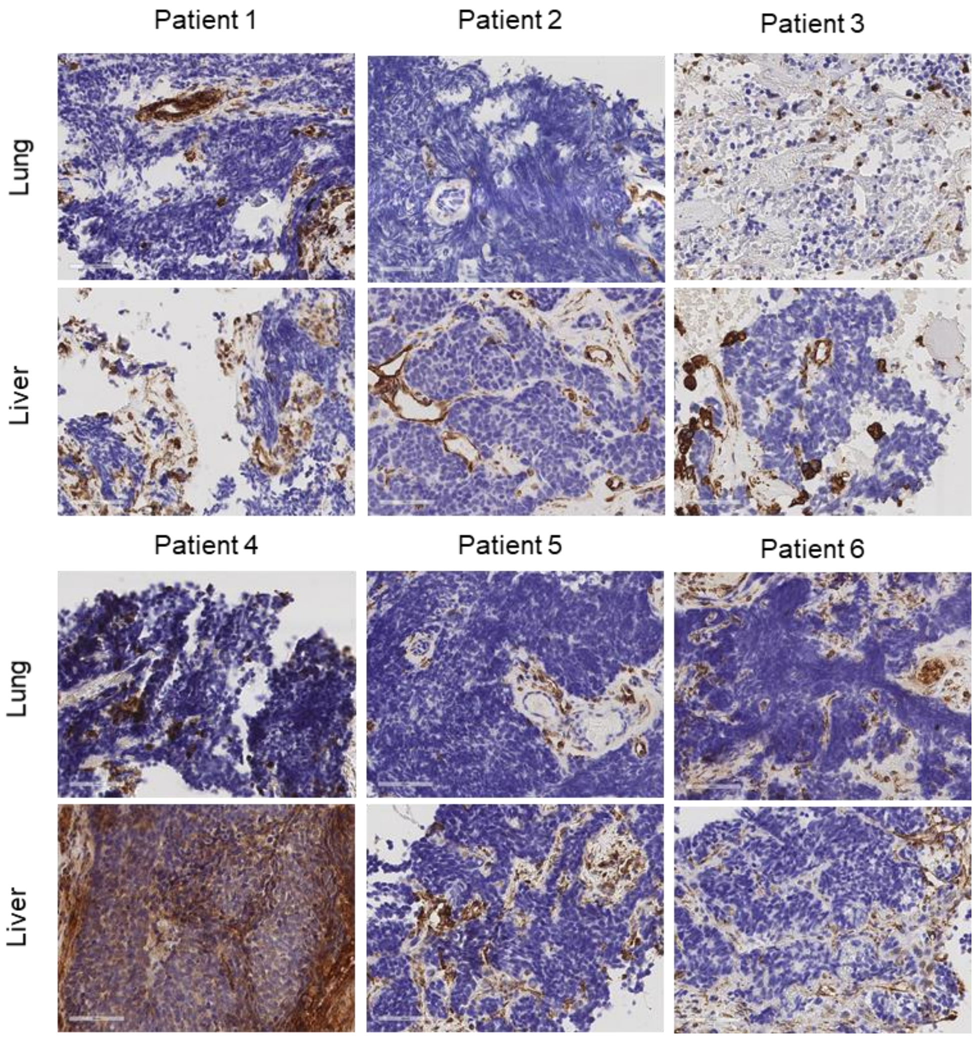

Figure S4. Vimentin expression in liver metastasis of SCLC patients.
FFPE lung tumor material of six SCLC patients and matched liver metastasis was analyzed for the expression of Vimentin. Bars indicate 60 µm.

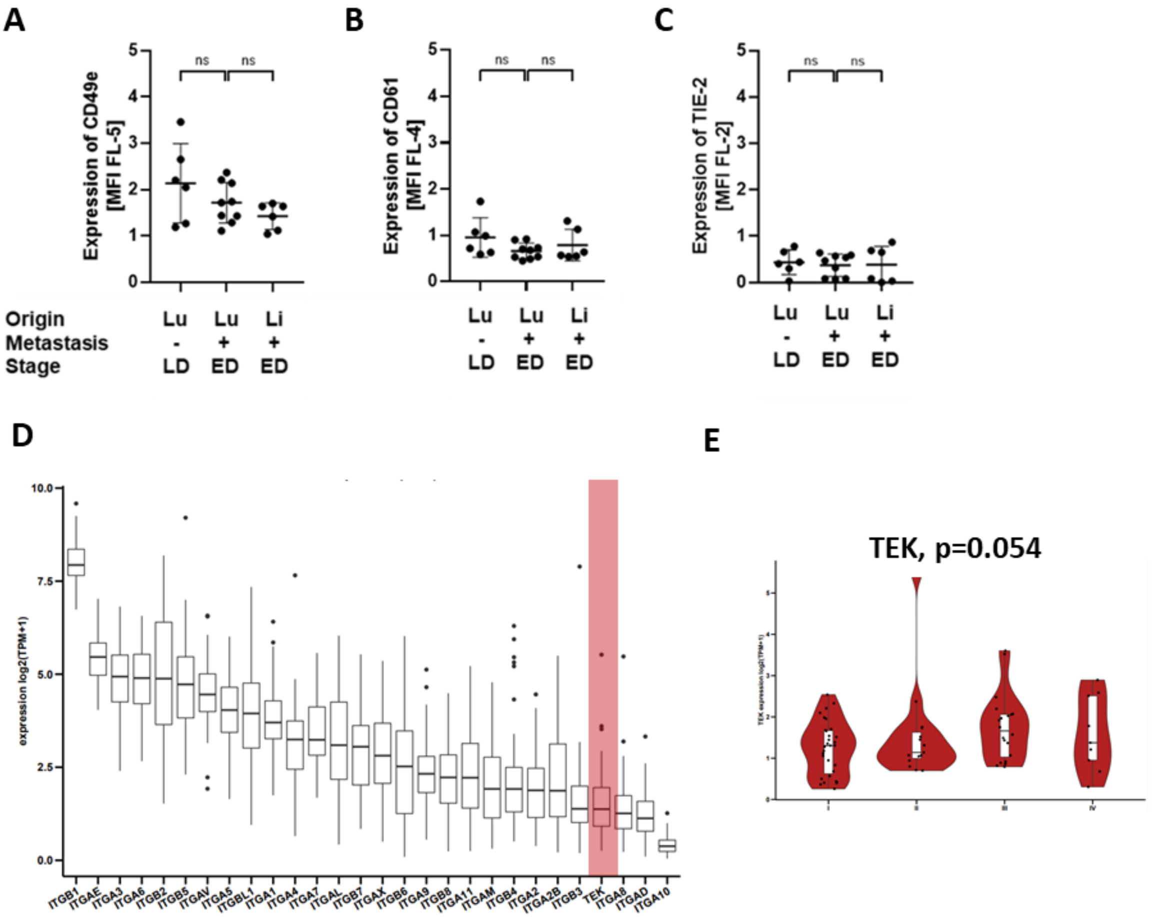

Figure S5. TIE-2 receptor expression is not altered upon extensive stage of disease on SCLC cells in vivo.

(A-C) Relative expression of the integrins CD49e (A) and CD61 (B) and TIE-2 (C) on SCLC tumor cells isolated from lung (n=6-9) and liver (n=6), normalized to IgG control, determined by flow cytometry. Origin of tumor cells, presence of metastasis and stage are indicated as limited stage of disease (LD) and extensive stage of disease (ED). Statistical analysis was done using the Student's t-test (ns, not significant; *, $p < 0.05$; **, $p < 0,01$;***, $p < 0,001$; error bars, SEM). (D) Human SCLC RNA Seq data (n=81) was analyzed regarding TEK (encoding for TIE-2) correlations. (E) Association between TEK expression and UICC tumor stage were tested using the non-parametric Jonckheere-Terpstra test (n=75).

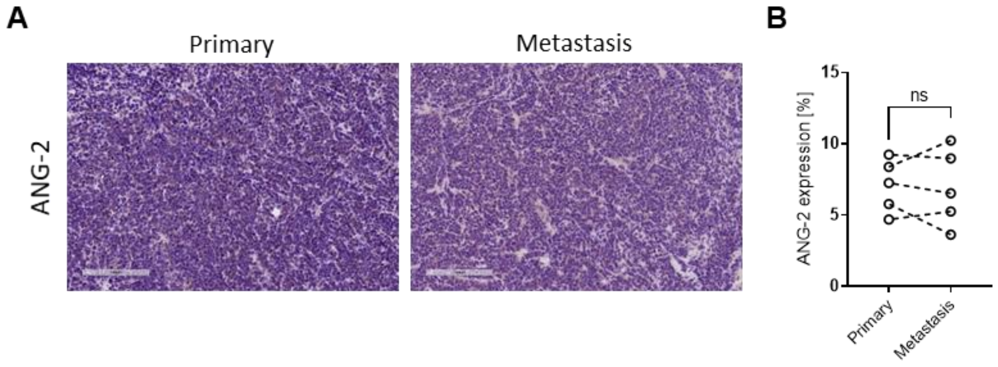

Figure S6. ANG-2 is not significantly increased in SCLC liver metastasis.
(A) Representative IHC results (n=5) for ANG-2 in matched murine tumors obtained from the primary SCLC and SCLC liver metastasis. Bars indicate 200 µm. (B) Quantification of protein expression using the threshold method of ImageJ in matched samples. Statistical analysis was done using the Student‘s t-test (ns, not significant; *, $p < 0.05$; **, $p < 0,01$;***, $p < 0,001$; error bars, SEM).

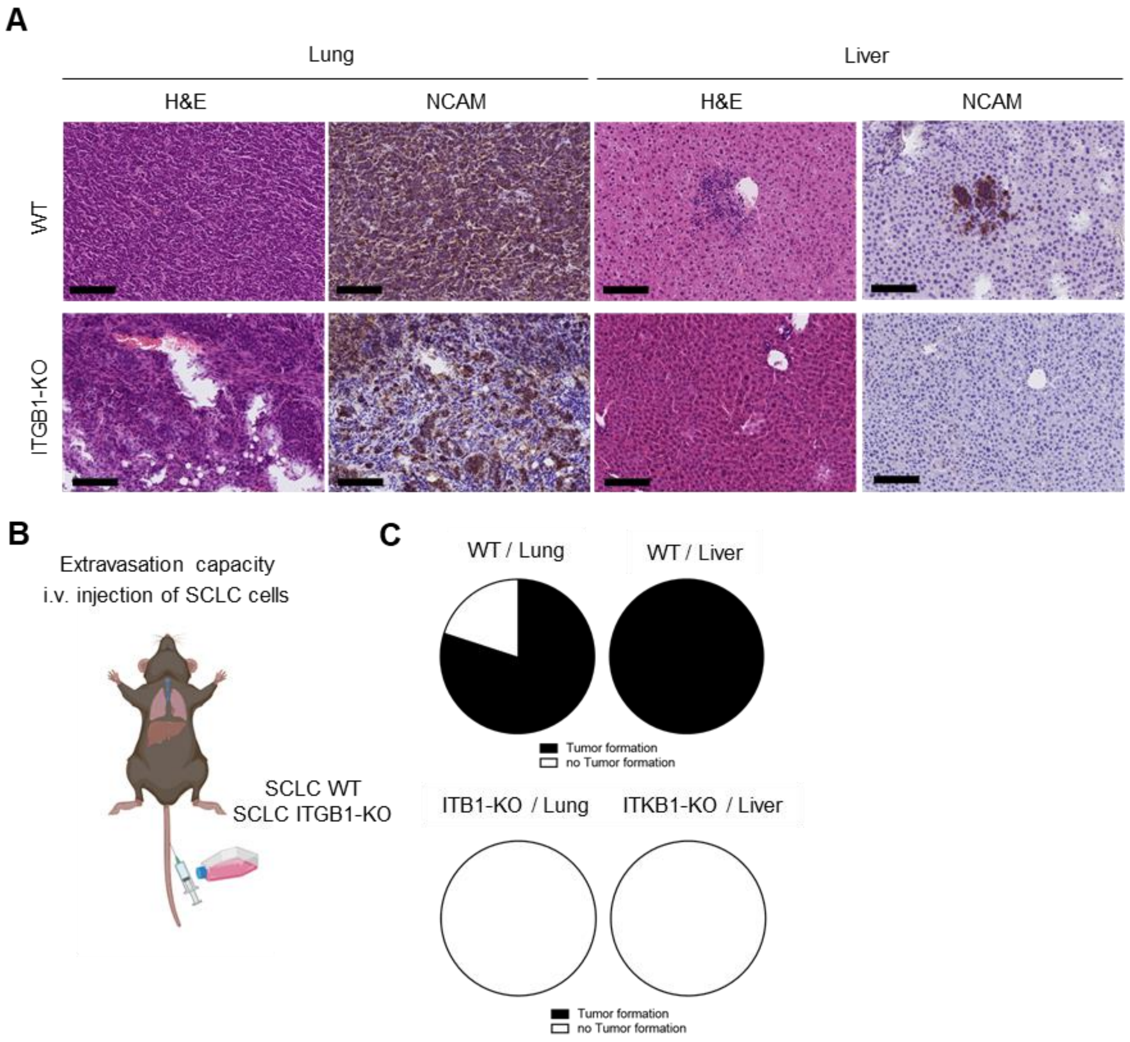

Figure S7. ITGB1 signaling is needed for extravasation of SCLC cells.
Representative images of H&E and NCAM IHC stain (n=5) of SCLC WT and ITGB1-KO orthotopically injected into the lung. Bars indicate 100 µm. (B) Scheme of i.v. injection of SCLC cells to determine extravasation capacity. Created with BioRender.com. (C) The capability of wildtype SCLC clones and ITGB1-KO to form tumors after i.v. injection was determined by IHC based on H&E and NCAM and quantified for lungs and livers, respectively (n=5).

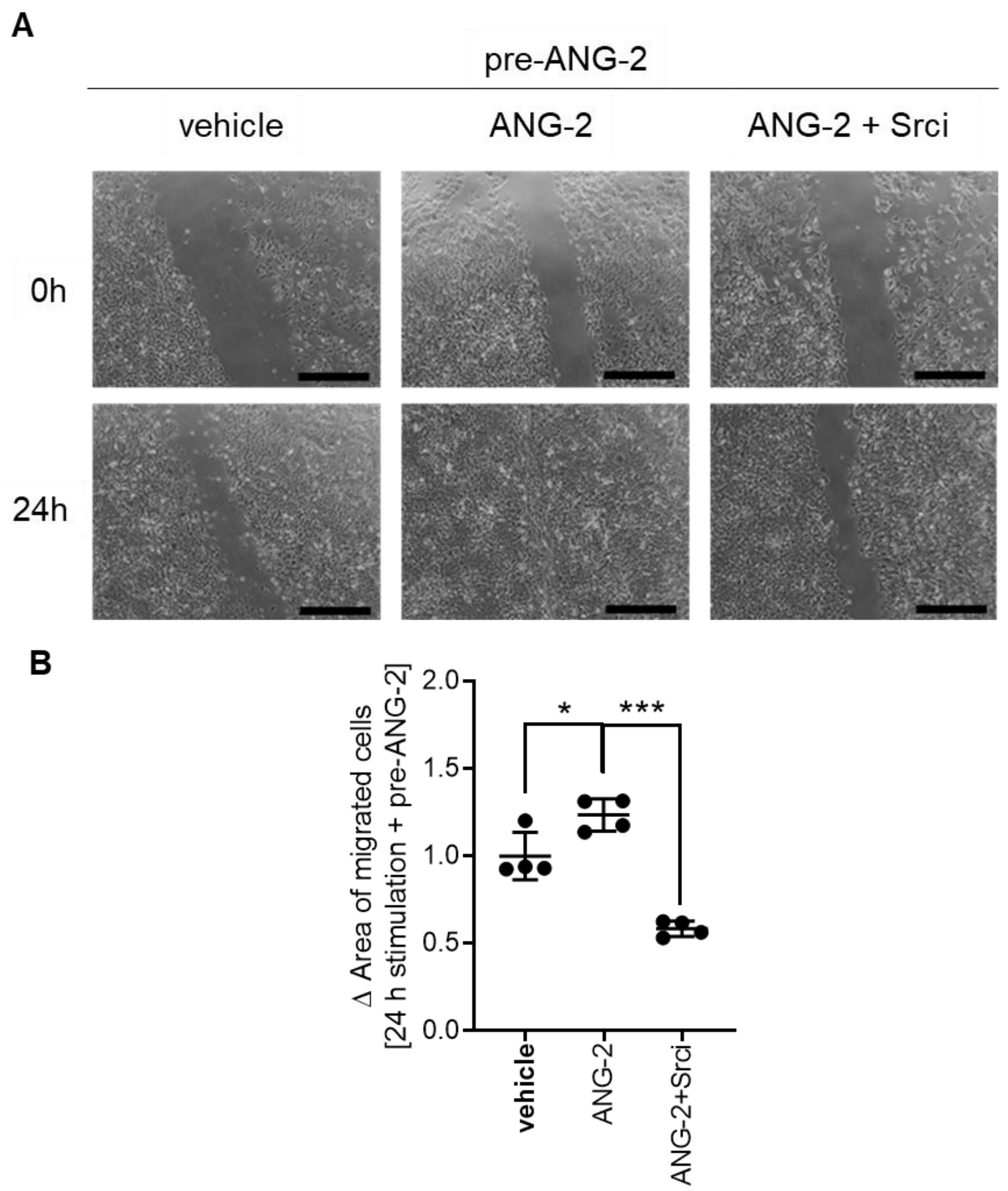

Figure S8. ANG-2-dependent ITGB1 mediated SCLC cell migration is regulated by SRC. (A+B) The area of migrated SCLC tumor cells isolated from liver metastasis pre-stimulated with Angiopoietin-2 for 24 h was determined by scratch assay. Cells were stimulated with Angiopoietin-2 and simultaneously treated with 100 nM Saracatinib for 24 h. Representative images out of three experiments. Statistical analysis was done using the Student's t-test (ns, not significant; *, $p < 0.05$; **, $p < 0,01$; ***, $p < 0,001$; error bars, SEM).

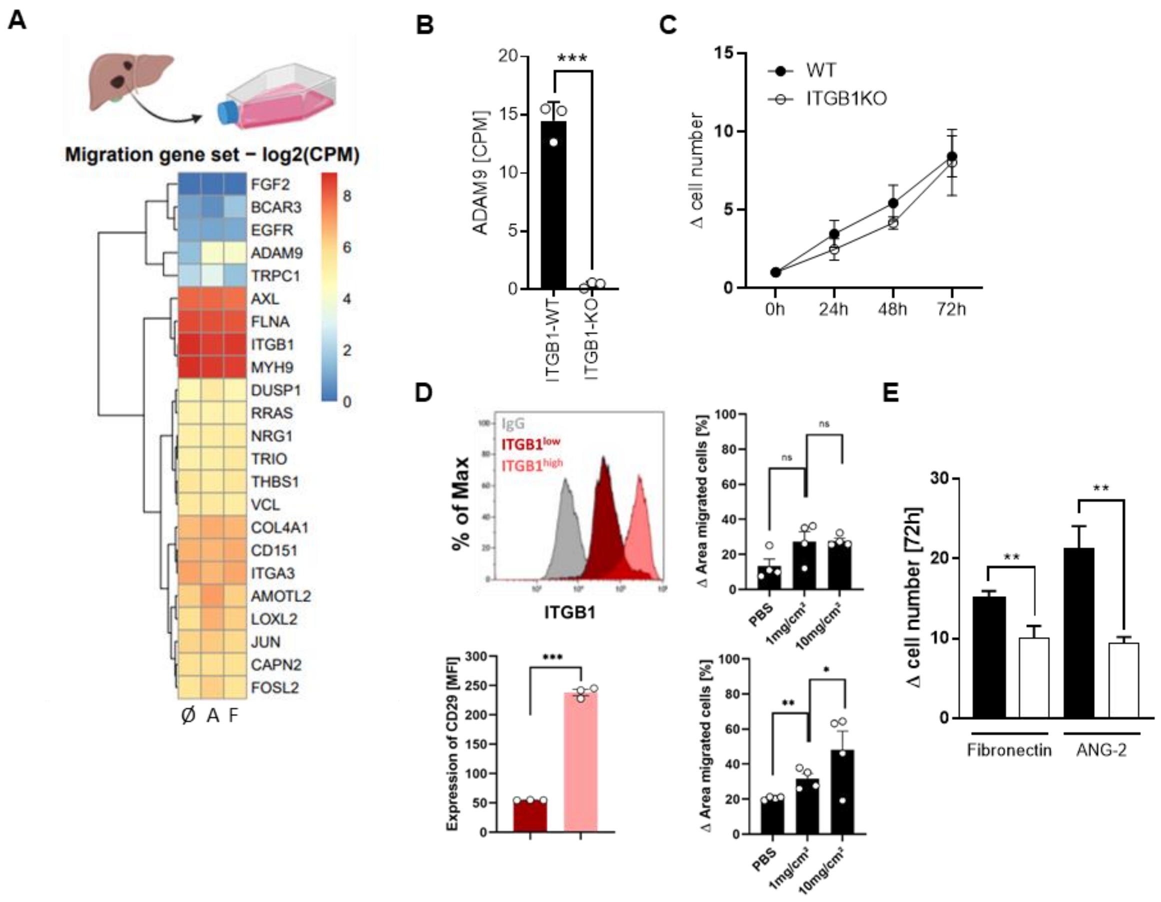

Figure S9. ITGB1 expression correlates with SCLC migration capacity and ANG-2 and Fibronectin provide similar transcripts associated to cell migration.

(A) SCLC liver metastasis cells (Scheme created with BioRender.com) were stimulated with Angiopoietin-2 “A” and Fibronectin “F” and compared to control “O” by RNASeq regarding migration genes. (B) ADAM9 counts per million (CPM) of normalized RNA Sequencing data of ITGB1 knock-outs (n=3) vs controls (n=3) are shown. (C) Cell counting assay for 72h of SCLC cells obtained from liver metastasis and ITGB1KO generated by CRISPR Cas9. One representative experiment out of three. (D) ITGB1 protein expression was determined by flow cytometry on generated murine SCLC cell lines 5 passages after generation. SCLC cell migration upon Fibronection stimulation for 24 h determined by scratch assay. Images after 0 h and 24 h were analyzed using ImageJ. (E) Cell counting after 72h stimulation with fibronectin or ANG-2 of SCLC cells obtained from liver metastasis and ITGB1KO generated by CRISPR Cas9. One representative experiment out of three. Statistical analysis was done using the Student‘s t-test (ns, not significant; *, $p < 0.05$; **, $p < 0,01$; ***, $p < 0,001$; error bars, SEM).

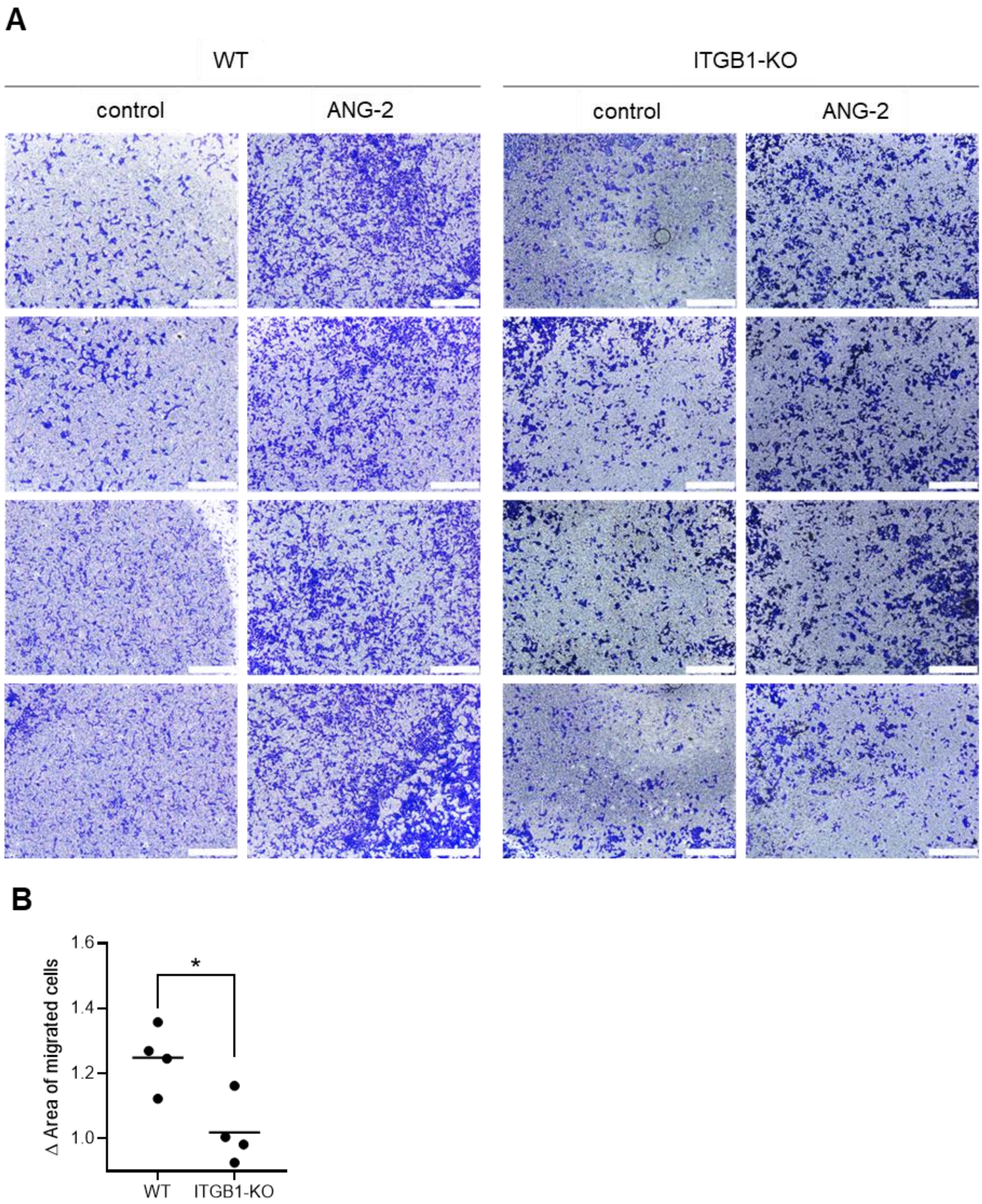

Figure S10. ITGB1 expression correlates invasion capacity.

(A) Representative pictures of WT clones and ITGB1-KO clones and quantification of four biological replicates determining SCLC cell invasion upon Angiopoietin-2 stimulation compared to control, measured by Boyden chamber method and crystal violet stain. (B) Quantification was performed using ImageJ. Statistical analysis was done using the Student‘s t-test (ns, not significant; *, $p < 0.05$; **, $p < 0,01$;***, $p < 0,001$; error bars, SEM).

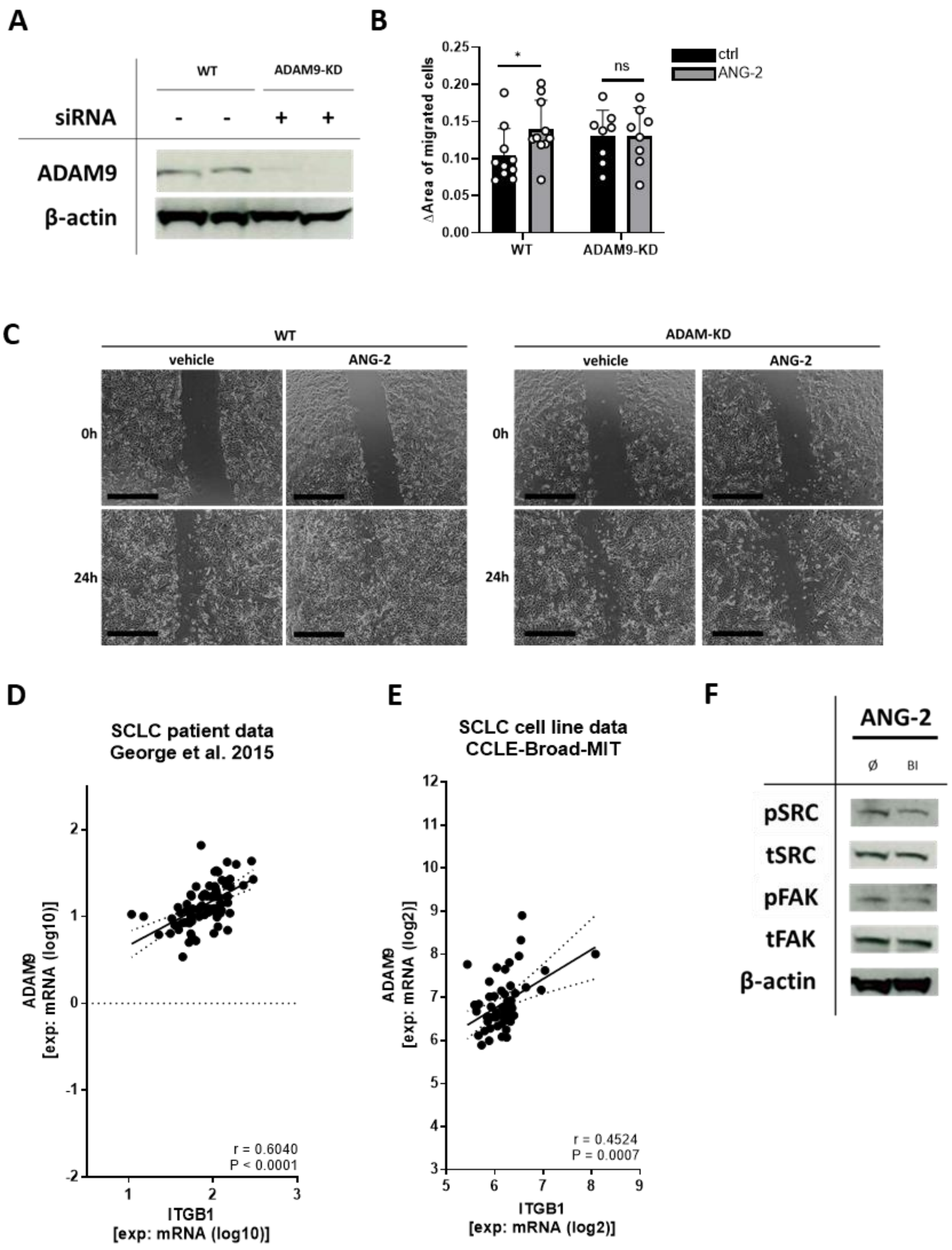

Figure S11. ADAM9 is responsible for SCLC cell migration and regulated downstream of ITGB1.

(A) ADAM9 is knocked down using two different siRNA. (B+C) The area of migrated SCLC tumor cells isolated from wild type liver metastasis with ADAM9 (WT) and without ADAM9 (ADAM9-KD) was determined by scratch assay. Cells were stimulated with Angiopoietin-2 (grey) for 24h and compared to control (ctrl). Representative images out of three experiments. Images after 0h and 24h were analyzed using ImageJ. (D) ADAM9 RNA expression data of SCLC patients (n=81) was correlated with ITGB1 gene expression using univariate analysis. Pearson correlation coefficient (r) and p-value are indicated. (E) ADAM9 RNA expression data of for human SCLC cell lines (n=64) in the SCLC CCLE-Broad-MIT data set provided by

CellMinerCDB was correlated with ITGB1 gene expression using univariate analysis. Pearson correlation coefficient (r) and p-value are indicated. (F) SCLC cells isolated from liver metastasis were pre-treated with ANG-2 for 20 min. Then intra-cellular FAK-SRC signaling was determined upon ANG-2 blocking (BI) for 1h by Western blot. Representative experiment out of three.

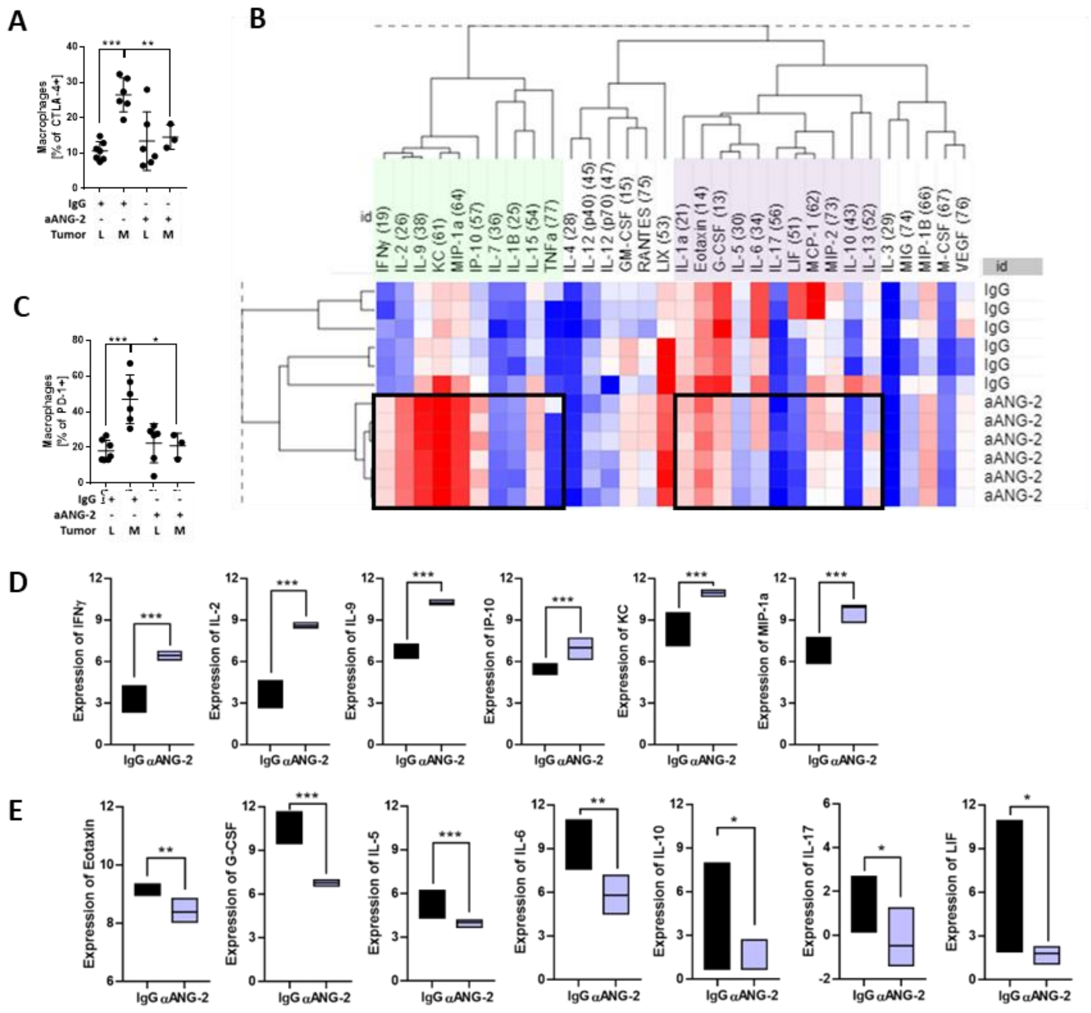

Figure S12. Anti-Angiopoietin-2 therapy promotes an anti-tumorigenic macrophage phenotype in vivo.

(A+B) SCLC-bearing mice were treated with IgG control (IgG; n=7) or anti-Angiopoietin-2 monotherapy (aANG-2; n=6). Upon detection of progressive disease based on mouse adapted RECIST v1.1 criteria determined by µCT, endpoint analysis was performed using flow cytometry. Primary tumor (L) and liver metastasis (M) were analyzed for fractions of CTLA-4 positive or PD-1 positive macrophages. Statistical analysis was done using the Student‘s t-test (ns, not significant; *, $p < 0.05$; **, $p < 0{,}01$;***, $p < 0{,}001$; error bars, SEM). (C) Serum samples of SCLC-bearing mice treated with IgG control or anti-Angiopoietin-2 monotherapy (aANG-2), were analyzed by a multiplex cytokine array. Cytokine concentrations were clustered using the Morpheus Broad Institute hierarchical clustering tool with the parameters Log2(1+X); Min 0; Max 15; Metric: 1- Pearson Correlation. Cytokine clustering revealed an M1-cluster (green) and an M2-cluster (violet). (D) Cytokines present in the M1-Cluster were analyzed for changes upon therapy. Significant changes upon anti-Angiopoietin-2 are indicated. (E) Cytokines present in the M2-Cluster were analyzed for changes upon therapy. Significant changes upon anti-Angiopoietin-2 are indicated. Statistical analysis was done using the Student‘s t-test (ns, not significant; *, $p < 0.05$; **, $p < 0{,}01$;***, $p < 0{,}001$; error bars, SEM).

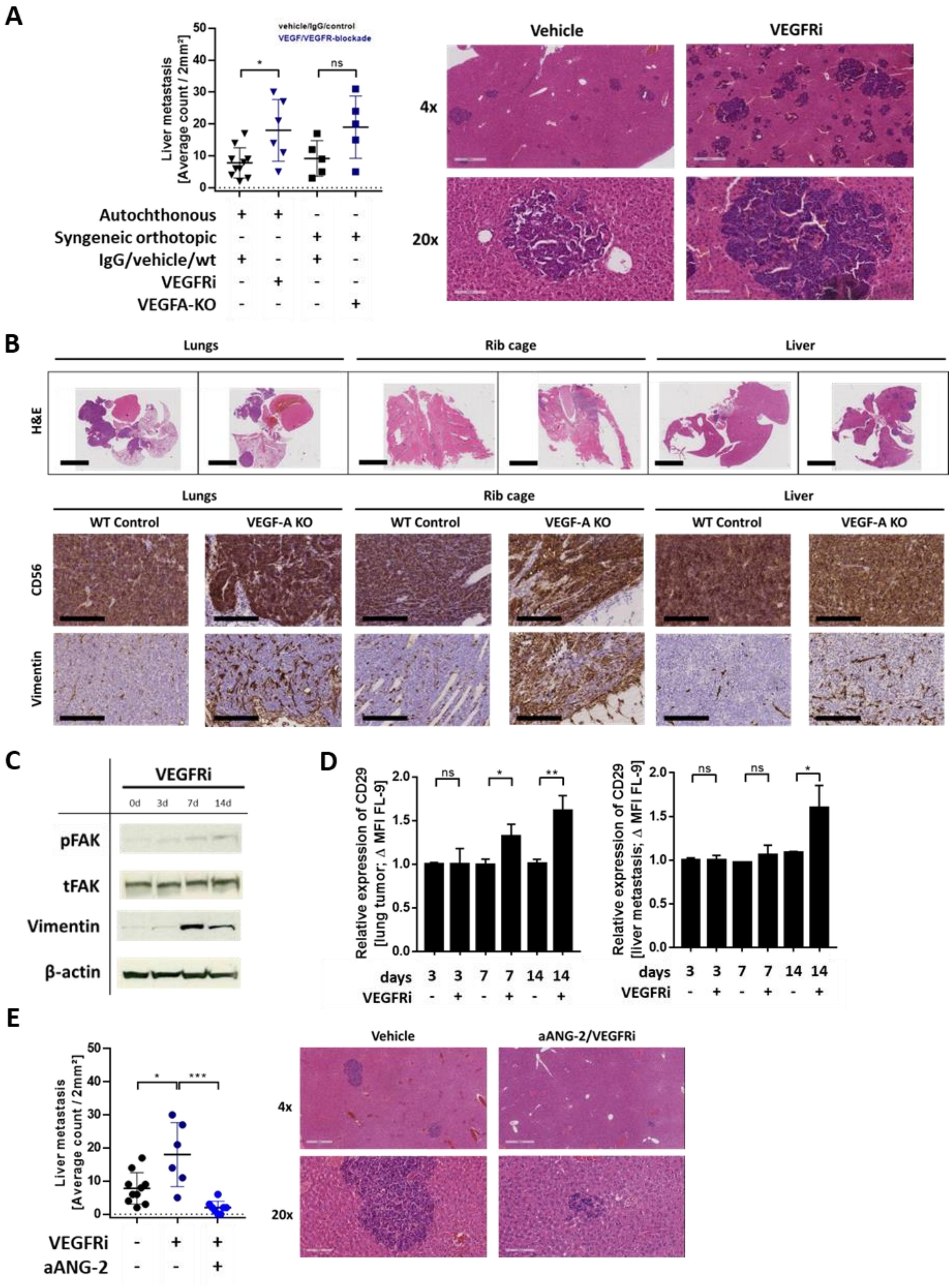

Figure S13. Targeting Angiopoietin-2-dependent ITGB1 signaling counteracts a VEGF/VEGFR-induced metastatic SCLC phenotype.

(A+B) Different models and conditions of VEGF/VEGFR signaling inhibition were analyzed for liver metastases. The average number of microscopic liver metastasis per $2mm^2$ was determined by scanned H&E slides in an autochthonous mouse model of SCLC and in a

syngeneic mouse model of SCLC, as indicated. VEGF/VEGFR signaling inhibition was achieved by two weeks of treatment with an VEGFR small molecule inhibitor, and CRISPR-Cas9 mediated knock-out of VEGF-A grown for two weeks after trans-thoracal injection of tumor cells. Bars indicate 100 µm. Representative images for relevant conditions are shown. Statistical analysis was done using the Student‘s t-test (ns, not significant; *, $p < 0.05$; **, $p < 0{,}01$;***, $p < 0{,}001$; error bars, SEM). The syngeneic model of wild-type SCLC and VEGF-A knock-out SCLC harbored rip cage metastasis and liver metastasis. Upper panel: H&E stain. Bars indicate 2 µm. Lower panel: Primary lung tumors and metastasis were analyzed for CD56 and Vimentin by IHC. Bars indicate 100 µm. (C) Intracellular FAK-SRC signaling was determined by western blot upon VEGFR inhibition of isolated and cultured SCLC liver metastasis cells. Representative experiment out of three. (D) Relative expression of ITGB1 (CD29) on isolated and cultured SCLC tumor cells (n=3) or from liver metastasis (n=3). Tumor cells were treated with the VEGFR inhibitor Vatalanib or vehicle for 14 consecutive days. Flow cytometry was performed on days 3, 7 and 14. Relative expression of CD29 is indicated as fold change, normalized to the expression level of vehicle control. (E) SCLC-bearing mice were treated for two weeks with the VEGFR inhibitior Vatalanib (n=6) alone or Vatalanib in combination with the anti-Angiopoietin-2 antibody (n=8) or with the corresponding vehicle control (n=10). Liver tissue was harvested and the average number of microscopic liver metastases per 2mm2 was counted by scanned H&E slides. Representative images for relevant conditions are shown. Bars indicate 100 µm. Statistical analysis was done using the Student‘s t-test (ns, not significant; *, $p < 0.05$; **, $p < 0{,}01$;***, $p < 0{,}001$; error bars, SEM).

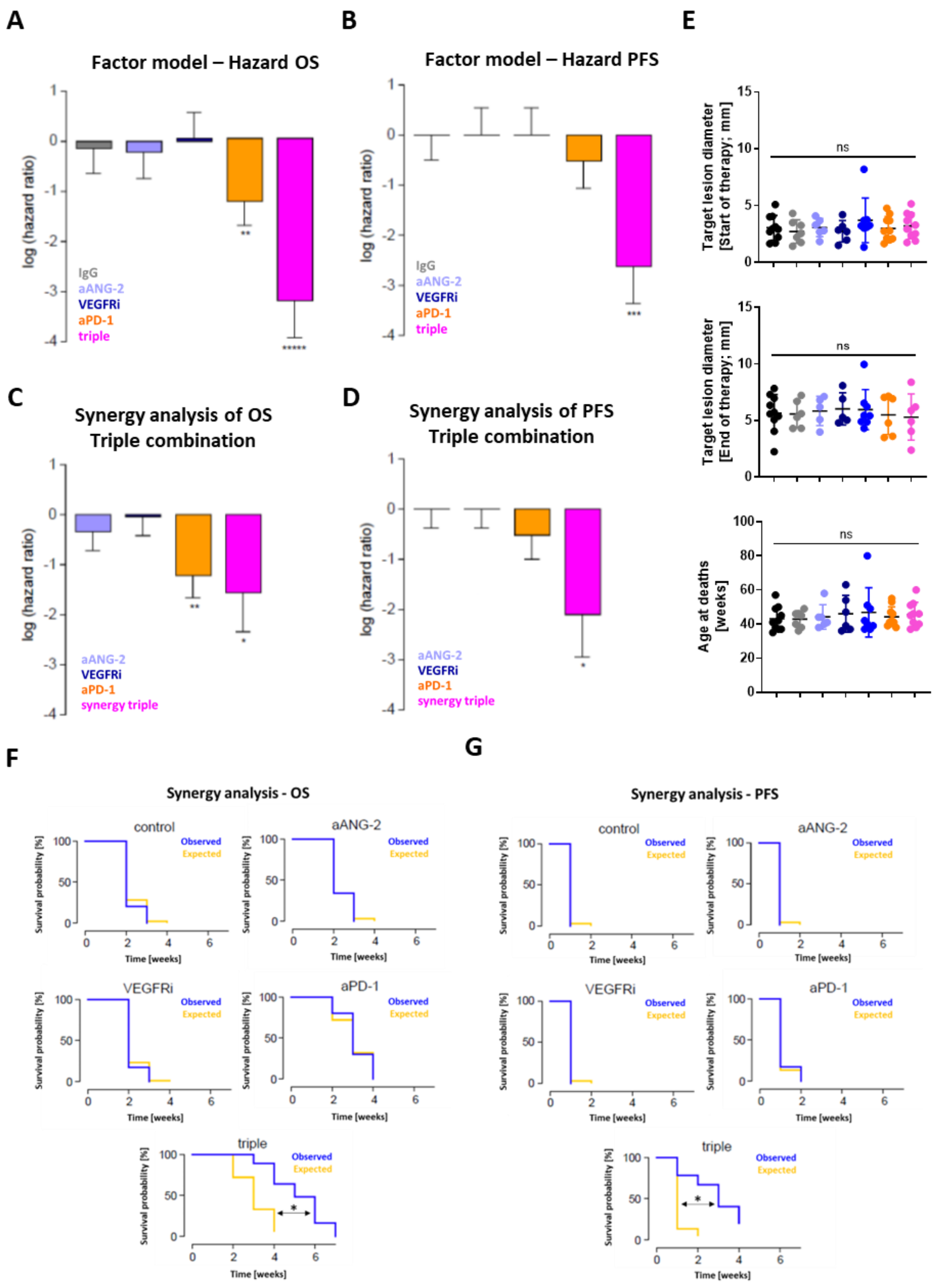

Figure S14. Combining Angiopoietin-2-, VEGFR- and PD-1 blockade synergistically prolongs survival of SCLC bearing mice.

SCLC-bearing mice were treated with vehicle control (black; n=10), IgG control (grey; n=7), VEGFR inhibitor monotherapy (VEGFRi, dark blue; n=6), anti-Angiopoietin-2 monotherapy (aANG-2, light blue; n=6), anti-PD-1 monotherapy (aPD-1, orange; n=10), and anti-

Angiopoietin-2/VEGFR inhibitor/anti–PD-1 triple combination therapy (triple, pink; n=10). (A) Log of the hazard ratio of the different therapy cohorts to show the suitability of the proportional hazard model for overall survival analysis. The received therapy is indicated by the color code. (B) Log of the hazard ratio of different therapy cohorts to show the suitability of the proportional hazard model for progression-free survival analysis. The received therapy is indicated by the color code. (C) Synergy analysis regarding overall survival of the triple combination with anti-Angiopoietin-2, VEGFR inhibitor and anti-PD-1 using the proportional hazard model and the R Survival package. P-values ≤ 0.05 are indicated as significant. ns – not significant. (D) Synergy analysis regarding progression-free survival of the triple combination with anti-Angiopoietin-2, VEGFR inhibitor and anti-PD-1 using the proportional hazard model and the R Survival package. P-values ≤ 0.05 are indicated as significant. ns – not significant. (E) Randomization of therapy groups in regards to target lesion diameter at the start of therapy, target lesion diameter at the end of therapy and the age at death. The received therapy is indicated by the color code. Statistical analysis was done using the Student‘s t-test (ns, not significant; *, $p < 0.05$; **, $p < 0,01$; ***, $p <0,001$; error bars, SEM). (F) Survival probability of the overall survival displaying the observed (blue) and expected (yellow) survival using the proportional hazard ratio model. (G) Survival probability of the progression-free survival displaying the observed (blue) and expected (yellow) survival using the proportional hazard ratio model. P-values ≤ 0.05 are indicated as significant. ns – not significant.

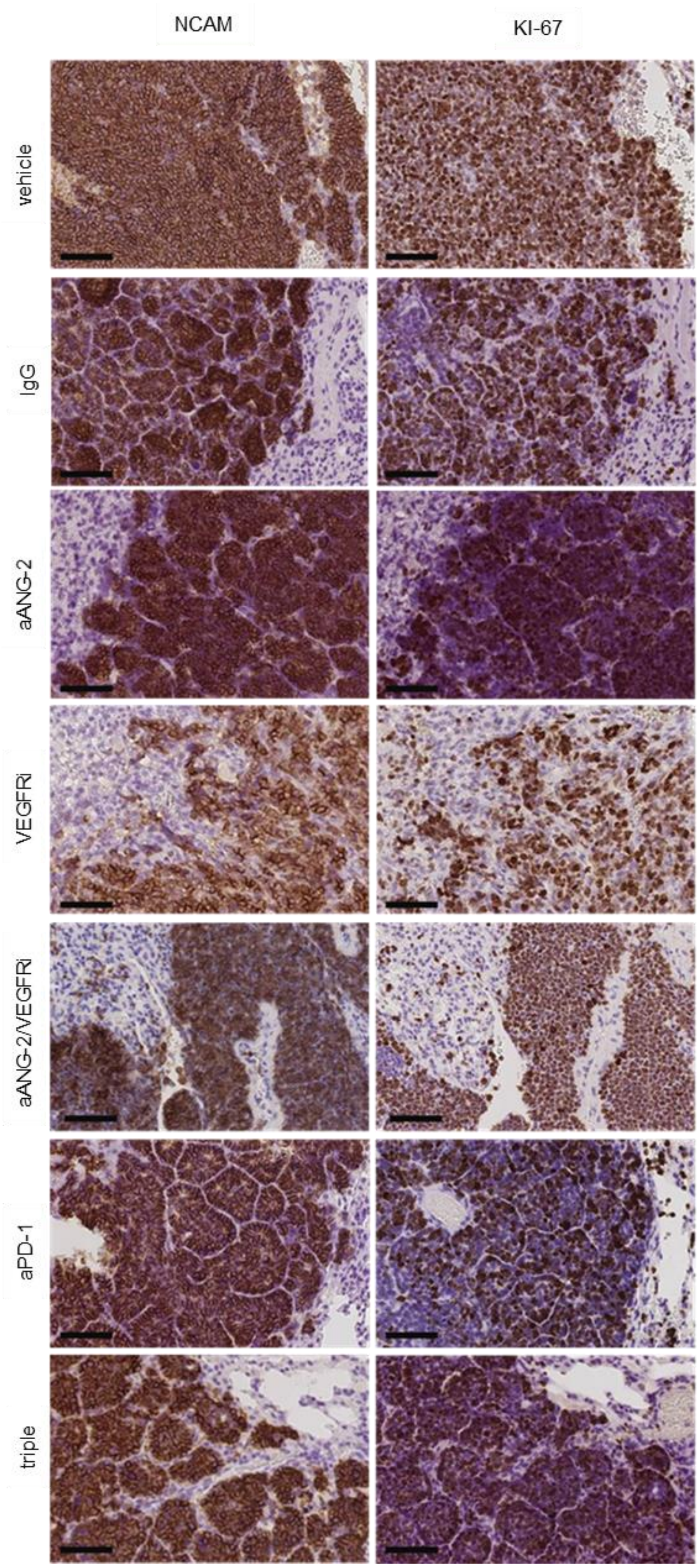

Figure S15. Murine primary SCLC tumors mimic the expression of NCAM and KI-67. SCLC-bearing mice were treated and upon detection of progressive disease based on mouse adapted RECIST v1.1 criteria determined by µCT, endpoint analysis was performed using IHC. NCAM and KI-67 IHC stains on FFPE SCLC tissue of primary lung tumors. Images were taken at 40x magnification. Bars indicate 50 µm. Representative images for each condition are shown:

(vehicle; n=9), IgG control (IgG; n=7), VEGFR inhibitor monotherapy (VEGFRi; n=6), anti-ANG-2 monotherapy (aANG-2; n=6), anti-ANG-2 and VEGFR inhibitor combination therapy (aANG-2/VEGFRi; n=5), anti–PD-1 monotherapy (aPD-1; n=6), and anti-ANG-2/VEGFR inhibitor/anti–PD-1 triple combination therapy (triple; n=8).

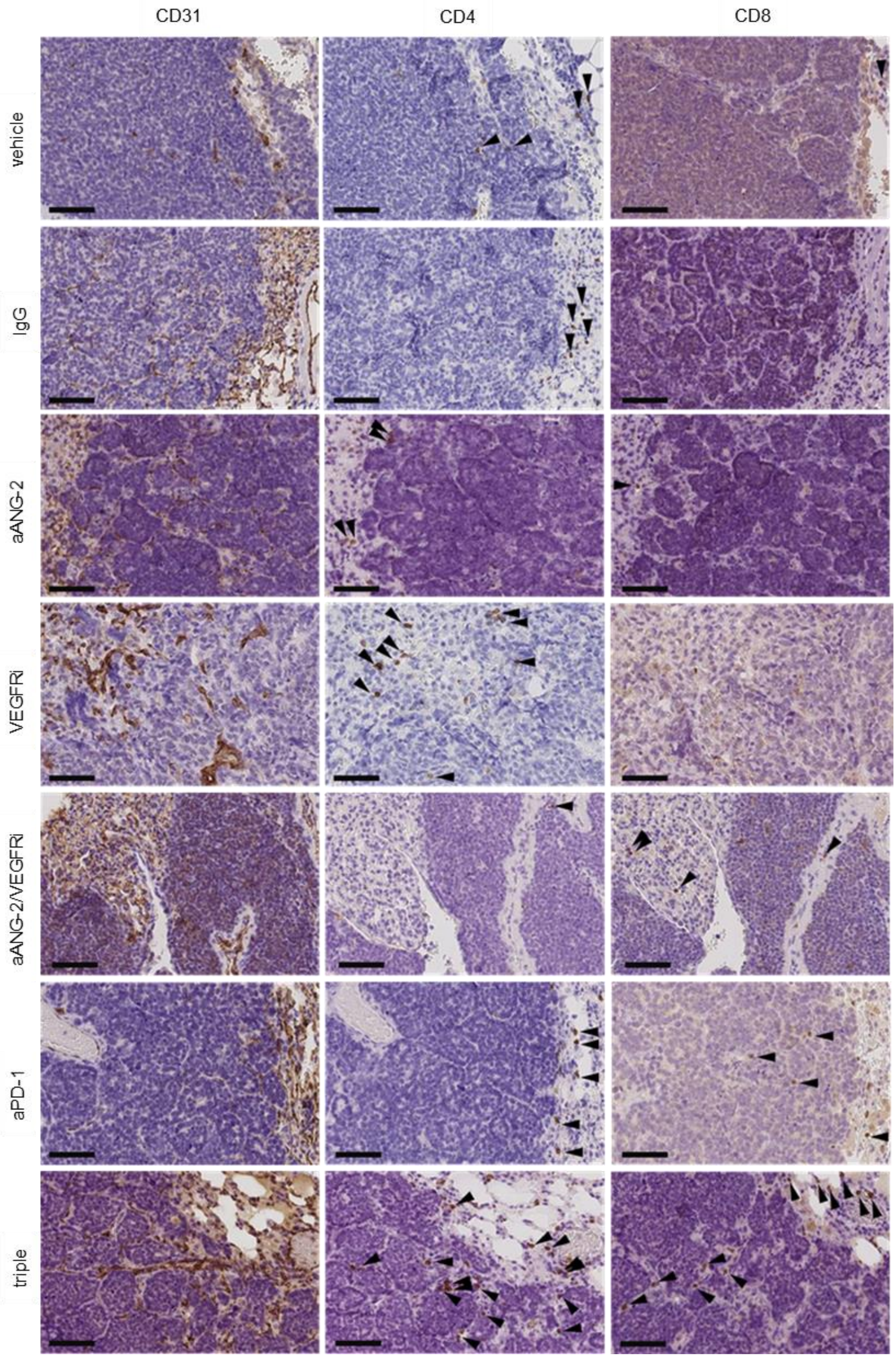

Figure S16. Murine primary SCLC tumors show increased T cell infiltration. SCLC-bearing mice were treated and upon detection of progressive disease based on mouse adapted RECIST v1.1 criteria determined by µCT, endpoint analysis was performed using IHC. CD31, CD4 and CD8 IHC stains on FFPE SCLC tissue of primary lung tumors. Images were taken at 40x magnification. Bars indicate 50 µm. Arrows indicate T cells of interest. Representative images for each condition are shown: (vehicle; n=9), IgG control (IgG; n=7), VEGFR inhibitor

monotherapy (VEGFRi; n=6), anti-ANG-2 monotherapy (aANG-2; n=6), anti-ANG-2 and VEGFR inhibitor combination therapy (aANG-2/VEGFRi; n=5), anti–PD-1 monotherapy (aPD-1; n=6), and anti-ANG-2/VEGFR inhibitor/anti–PD-1 triple combination therapy (triple; n=8).

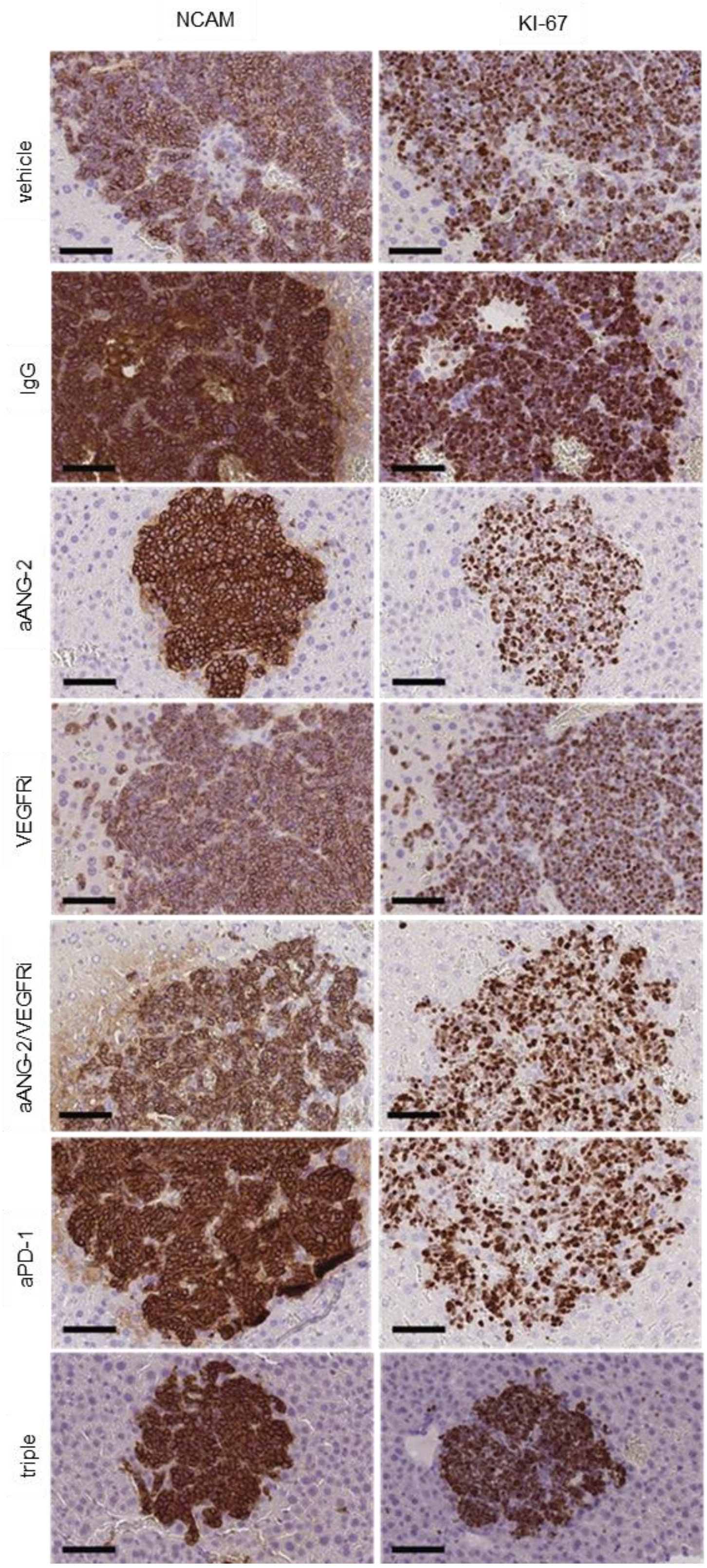

Figure S17. Murine SCLC liver metastases mimic the expression of NCAM and KI-67. SCLC-bearing mice were treated and upon detection of progressive disease based on mouse adapted RECIST v1.1 criteria determined by µCT, endpoint analysis was performed using IHC. NCAM and KI-67 IHC stains on FFPE SCLC tissue of SCLC liver metastases. Images were taken at 40x magnification. Bars indicate 50 µm. Representative images for each condition are shown: vehicle (n=7), IgG control (IgG; n=6), VEGFR inhibitor monotherapy (VEGFRi; n=6),

anti-ANG-2 monotherapy (aANG-2; n=3), anti-ANG-2 and VEGFR inhibitor combination therapy (aANG-2/VEGFRi; n=5), anti–PD-1 monotherapy (aPD-1; n=6), and anti-ANG-2/VEGFR inhibitor/anti–PD-1 triple combination therapy (triple; n=5).

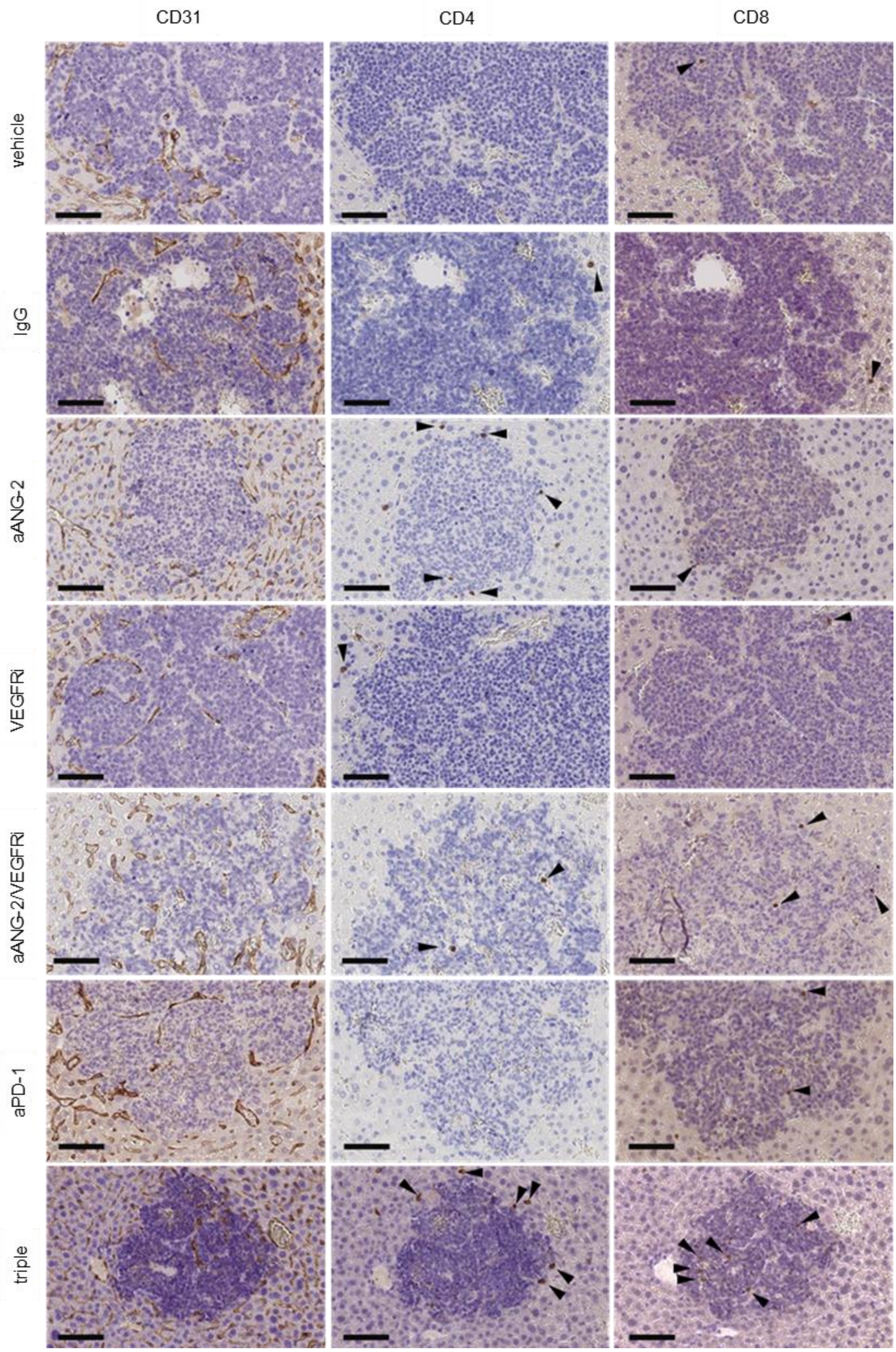

Figure S18. Murine SCLC liver metastases show increased T cell infiltration.
SCLC-bearing mice were treated and upon detection of progressive disease based on mouse adapted RECIST v1.1 criteria determined by µCT, endpoint analysis was performed using IHC. CD31, CD4 and CD8 IHC stains on FFPE SCLC tissue of SCLC liver metastases. Images were taken at 40x magnification. Bars indicate 50 µm. Arrows indicate T cells of interest. Representative images for each condition are shown: vehicle (n=7), IgG control (IgG; n=6),

VEGFR inhibitor monotherapy (VEGFRi; n=6), anti-ANG-2 monotherapy (aANG-2; n=3), anti-ANG-2 and VEGFR inhibitor combination therapy (aANG-2/VEGFRi; n=5), anti–PD-1 monotherapy (aPD-1; n=6), and anti-ANG-2/VEGFR inhibitor/anti–PD-1 triple combination therapy (triple; n=5).

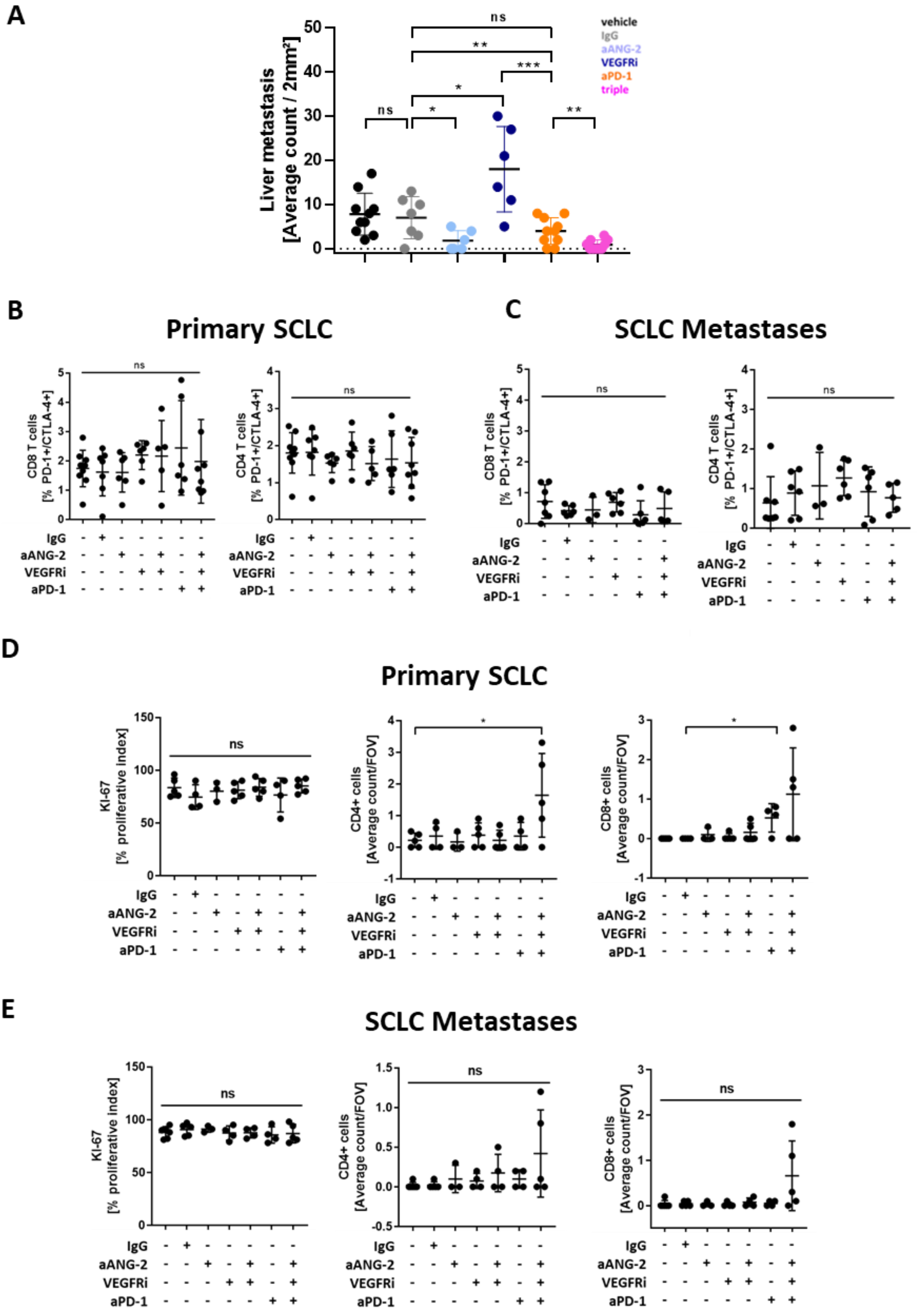

Figure S19. Liver metastases mimic primary SCLC regarding T cell exhaustion.
SCLC-bearing mice were treated with vehicle control (vehicle; n=9), IgG control (IgG; n=7), VEGFR inhibitor monotherapy (VEGFRi; n=6), anti-Angiopoietin-2 monotherapy (aANG-2; n=6), anti–PD-1 monotherapy (aPD-1; n=6), and anti-Angiopoietin-2/VEGFR inhibitor/anti–PD-1 triple combination therapy (triple; n=8). Upon detection of progressive disease based on

mouse adapted RECIST v1.1 criteria determined by µCT, (A) Liver tissue was harvested and the average number of microscopic liver metastases per $2mm^2$ was counted by three presentative scanned H&E slides. (B) The fraction of PD-1 and CTLA-4 double positive CD8+ and CD4+ T-cells was determined from primary SCLC lung tissue by flow cytometry. (C) The fraction of PD-1 and CTLA-4 double positive CD8+ and CD4+ T-cells was determined from SCLC liver metastases tissue by flow cytometry. (D) The KI-67 proliferative index and infiltrating CD4+ and CD8+ T cells were determined by IHC quantification per field of view (FOV) from primary SCLC lung tissue. (E) The KI-67 proliferative index and infiltrating CD4+ and CD8+ T cells were determined by IHC quantification per field of view (FOV) from SCLC liver metastases tissue. Statistical analysis was done using the Student‘s t-test (ns, not significant; *, $p < 0.05$; **, $p < 0,01$; ***, $p <0,001$; error bars, SEM).
