# Supplementary figures and images for: Blocking the angiopoietin-2–dependent integrin β-1 signaling axis abrogates small cell lung cancer invasion and metastasis
Source: JCI Insight. 2024 May 22;9(10):e166402. doi: 10.1172/jci.insight.166402 (PMC11141935; doi:10.1172/jci.insight.166402)

Figure 7 D

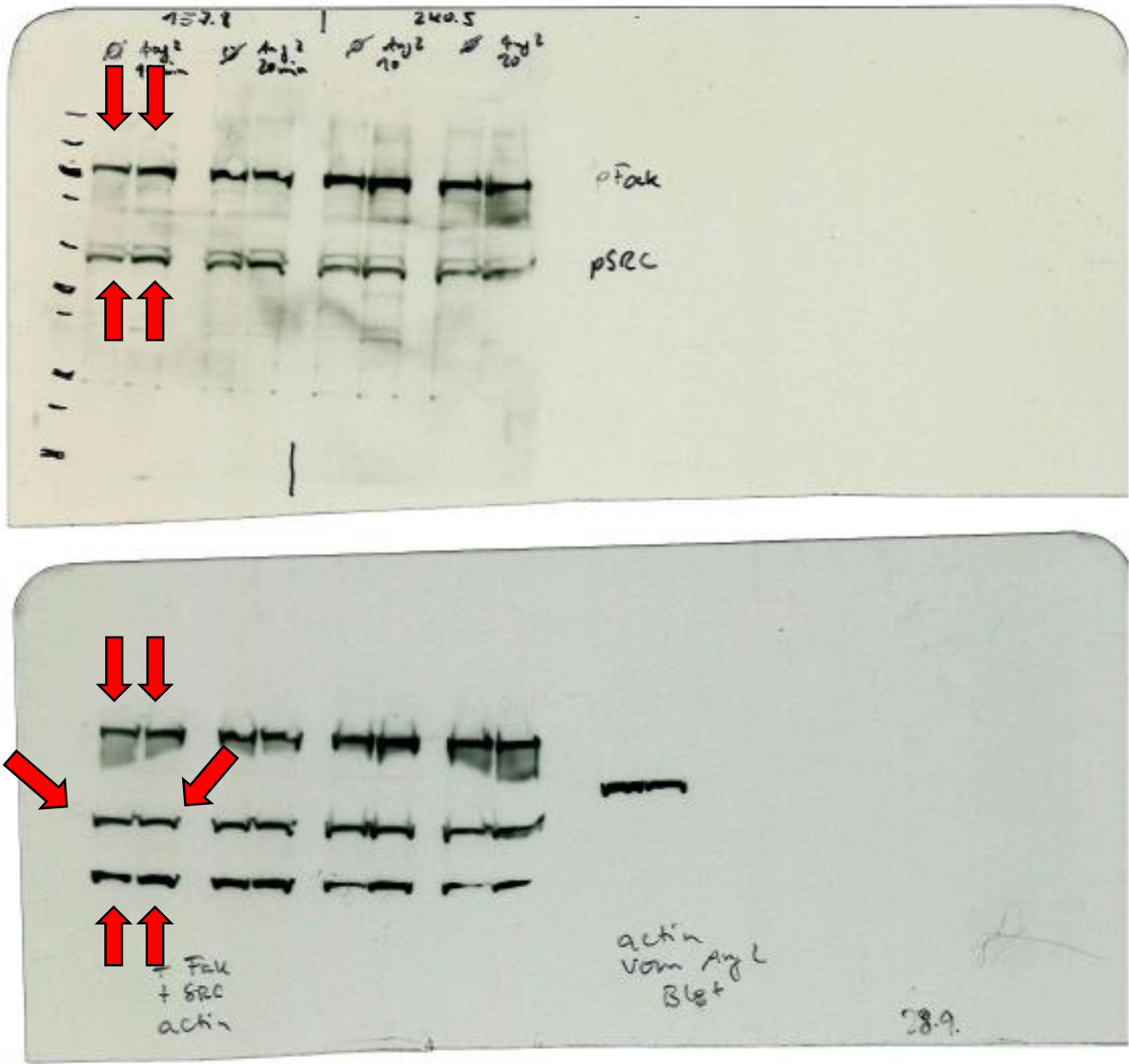

Figure 7F

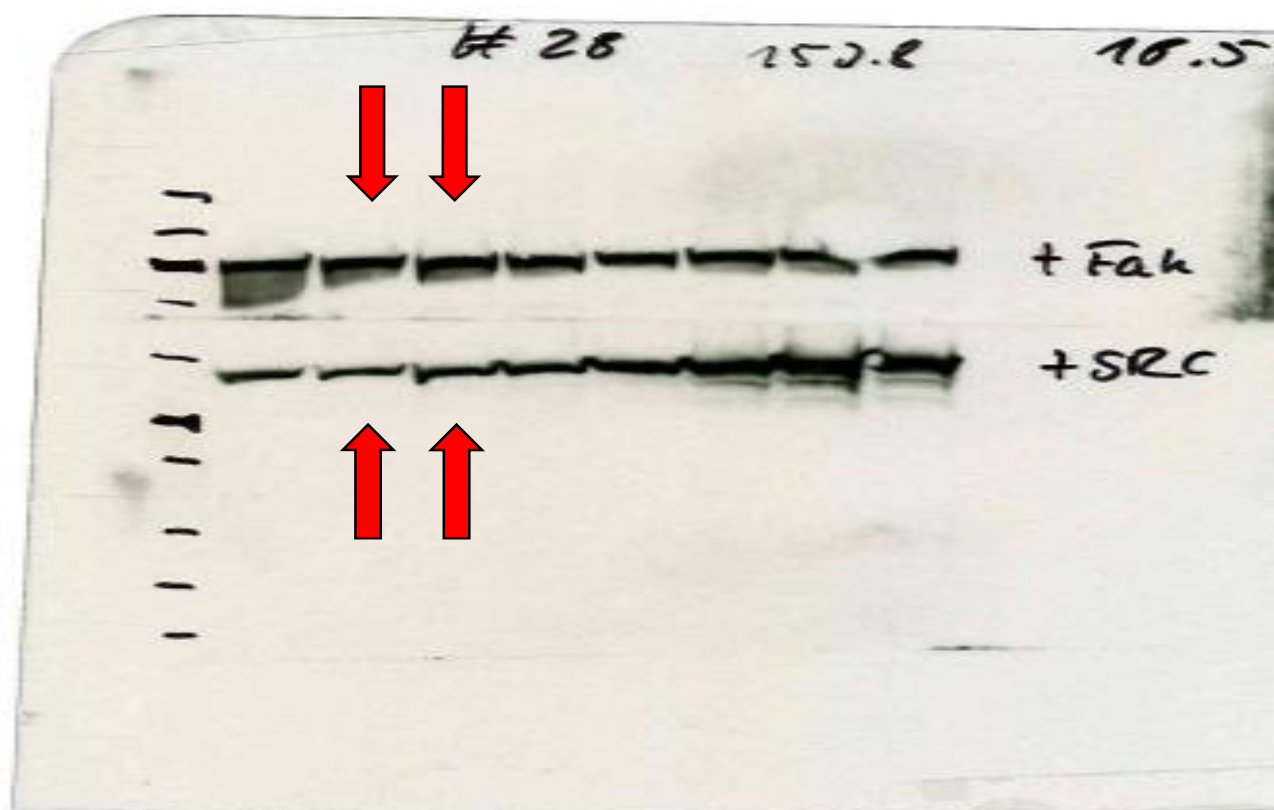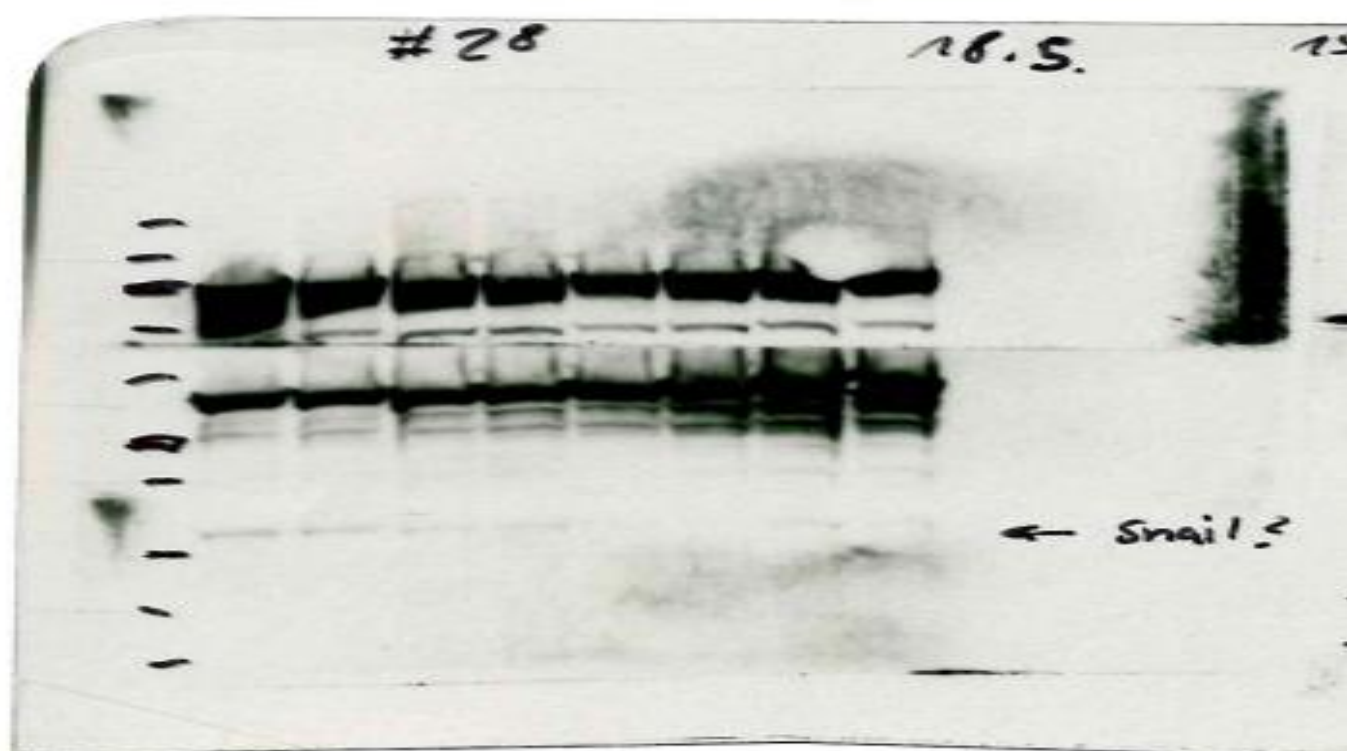

Figure 7F

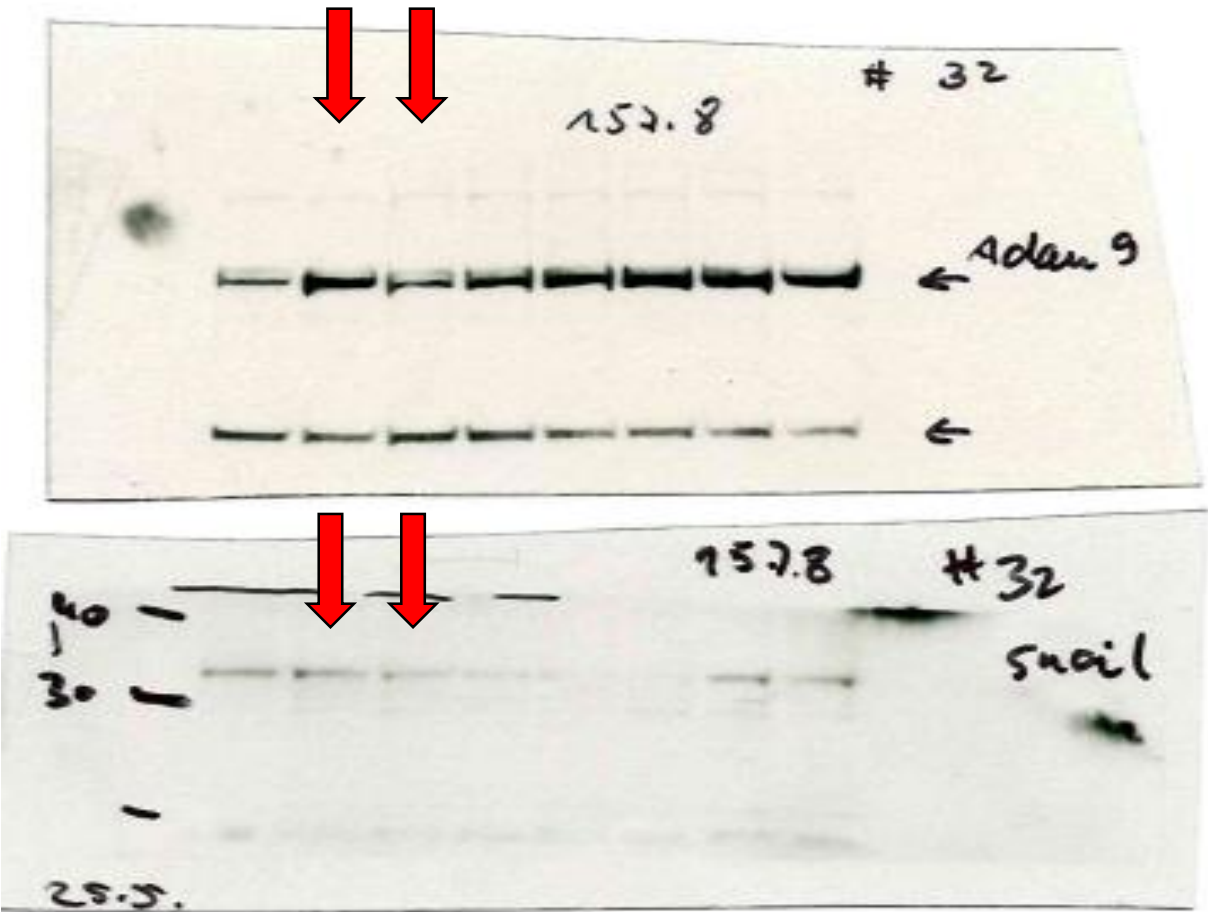

Figure 7F

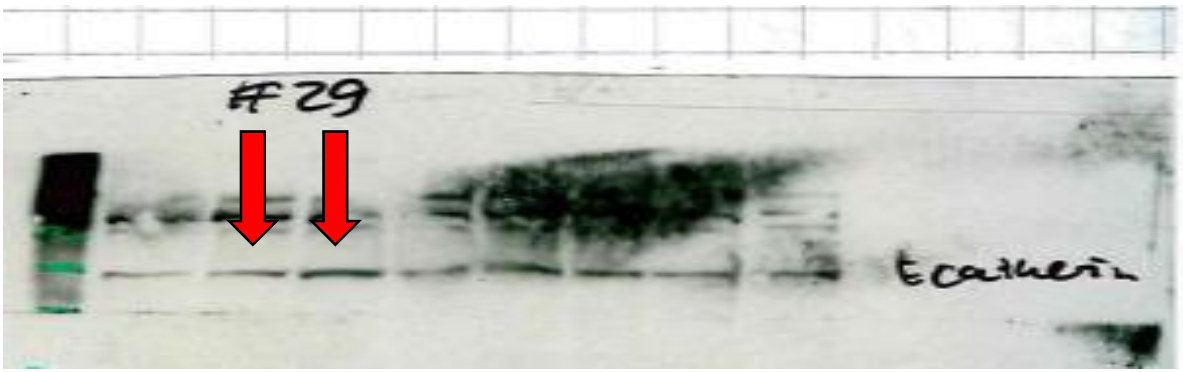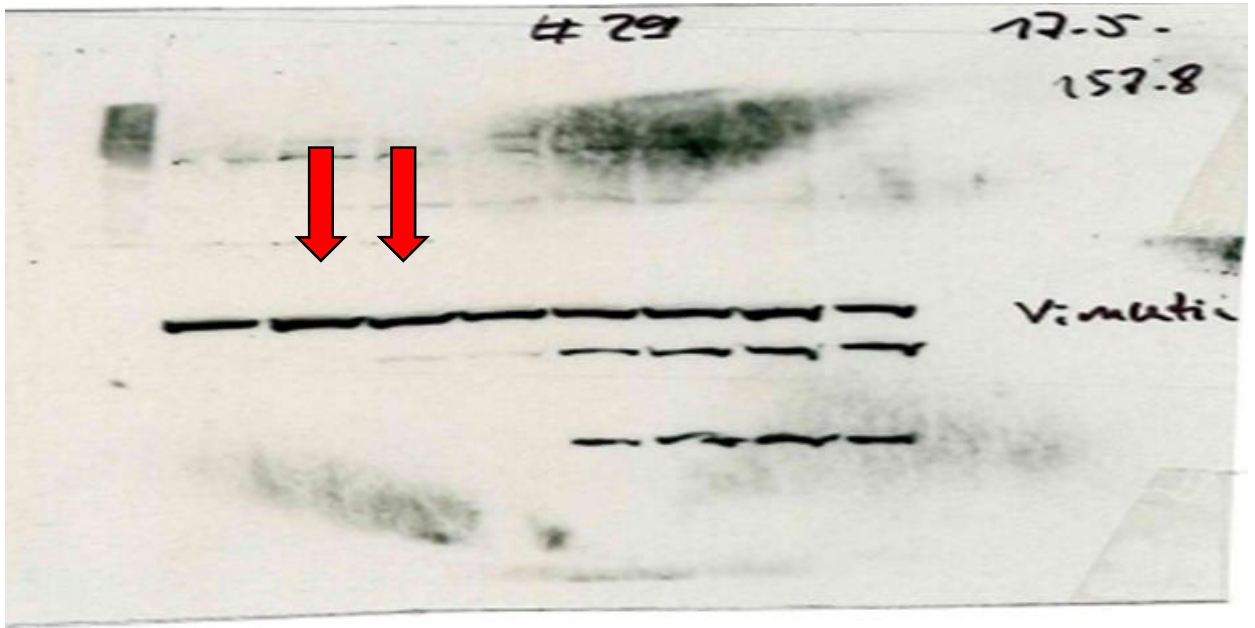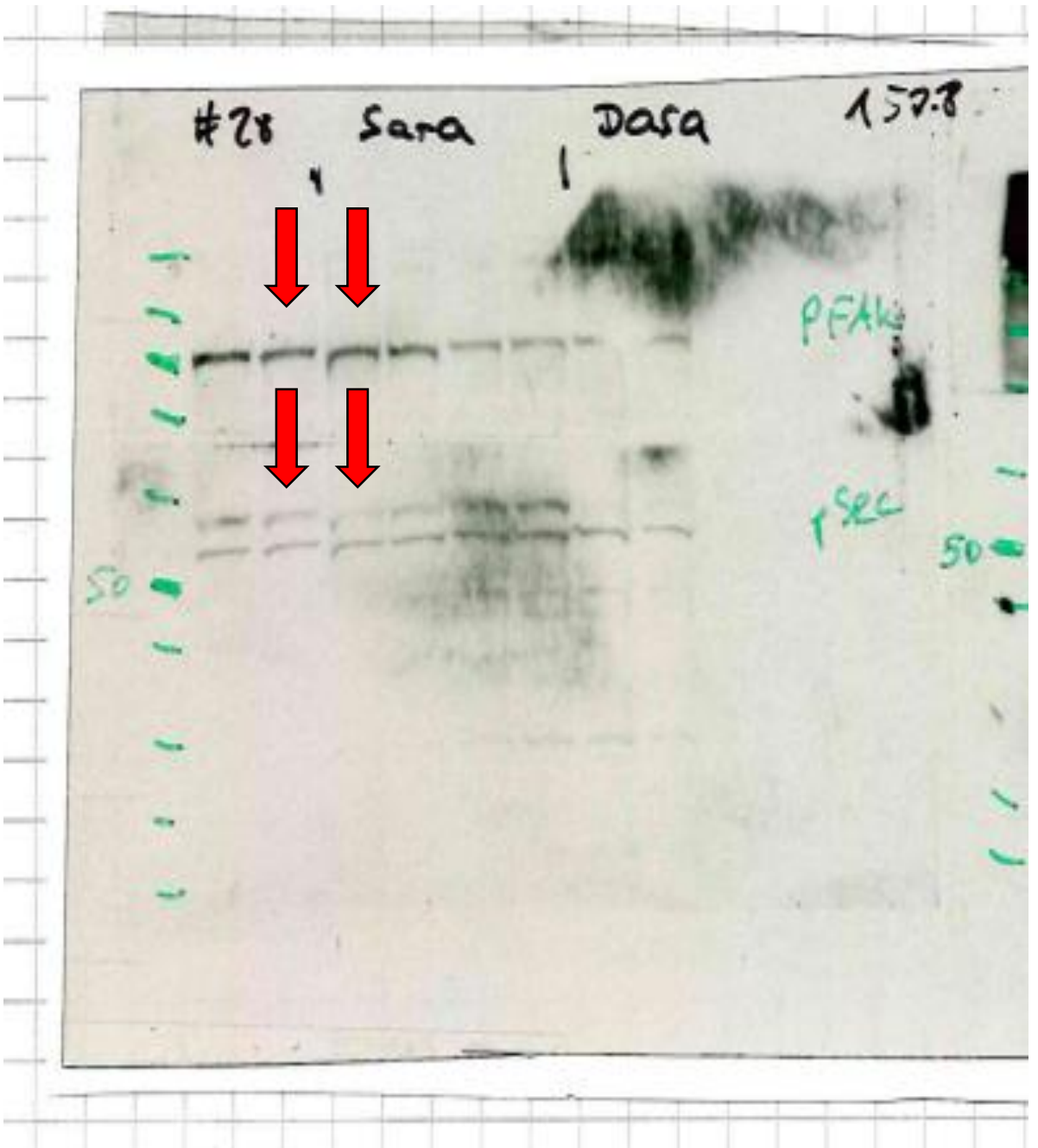

Figure 7F

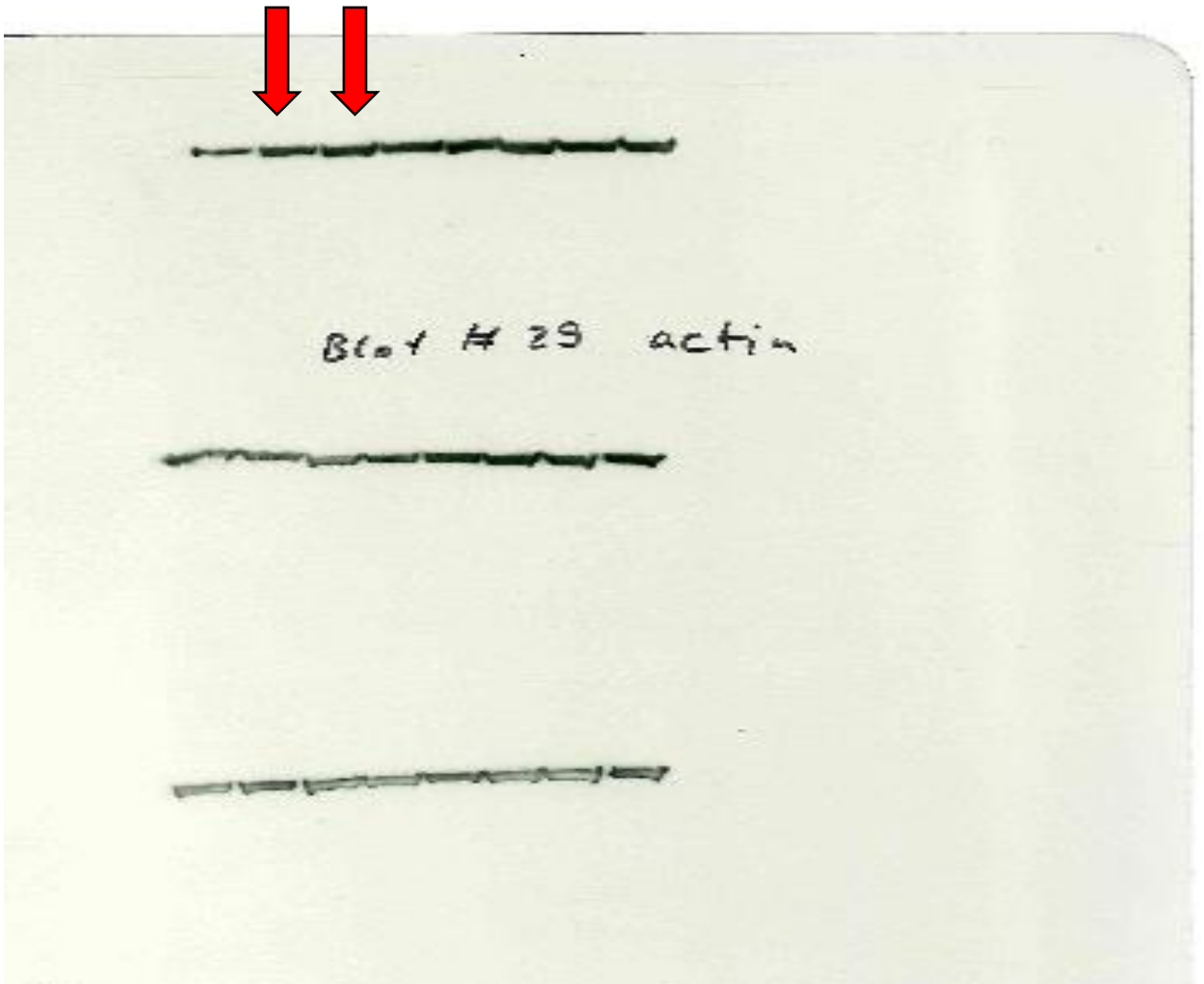

Figure S11A

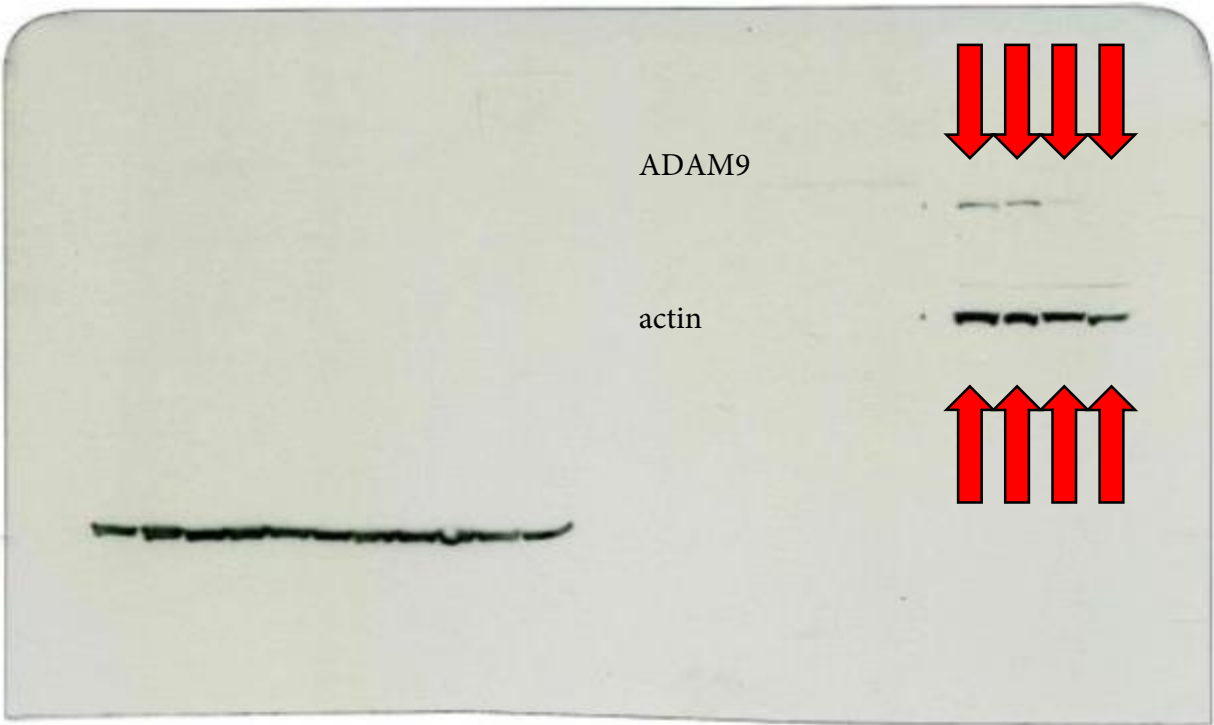

Figure S11F

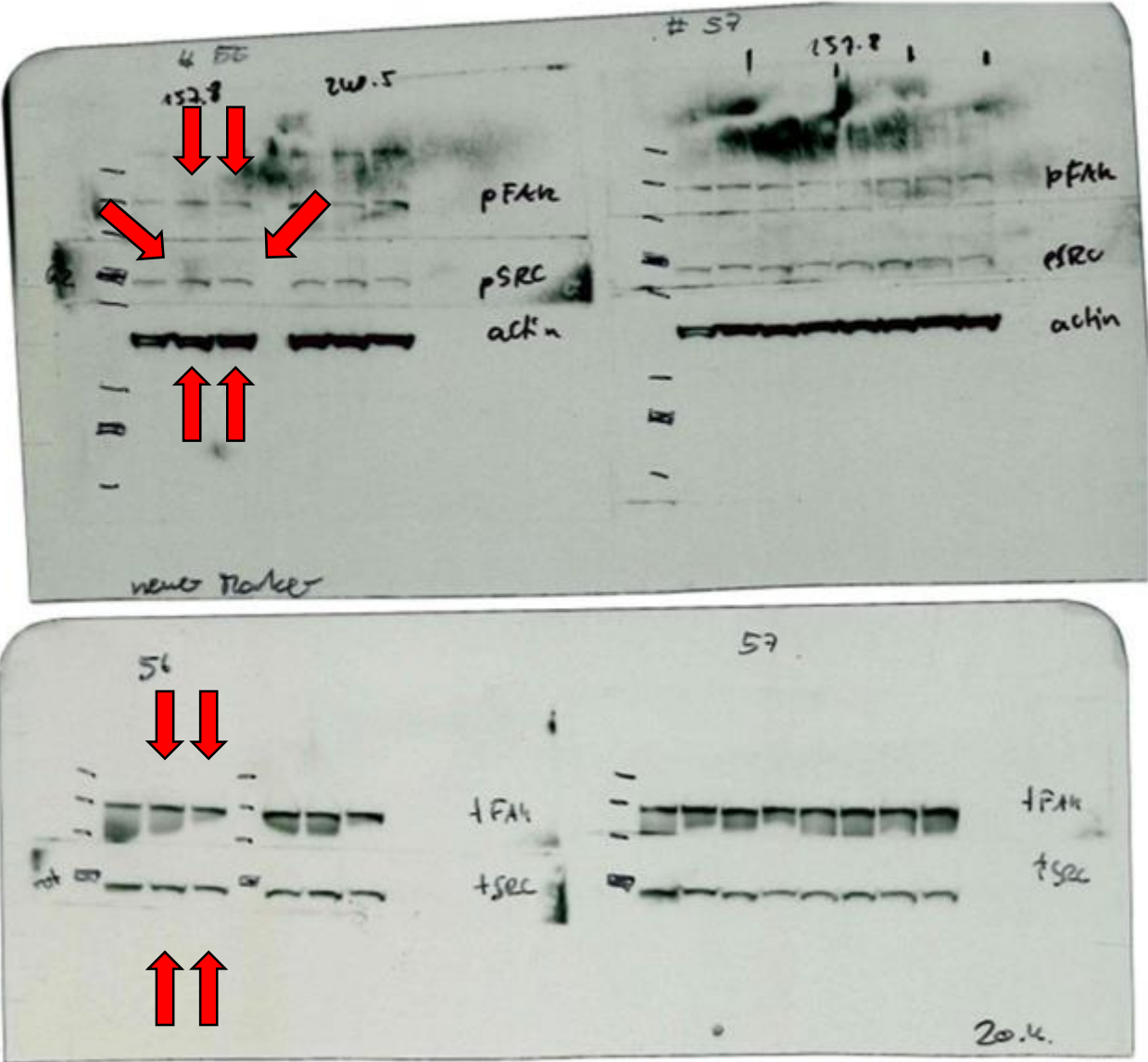

Figure S13C

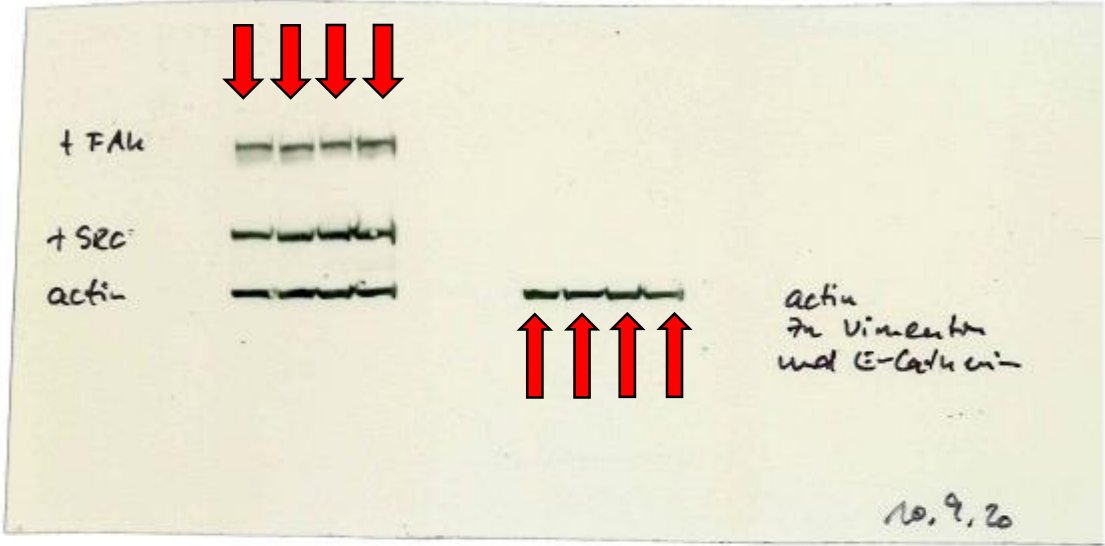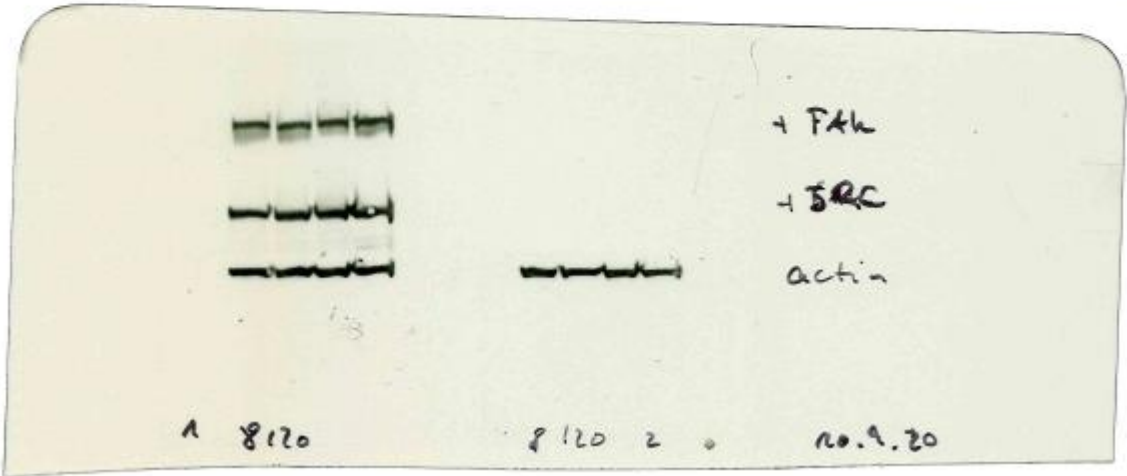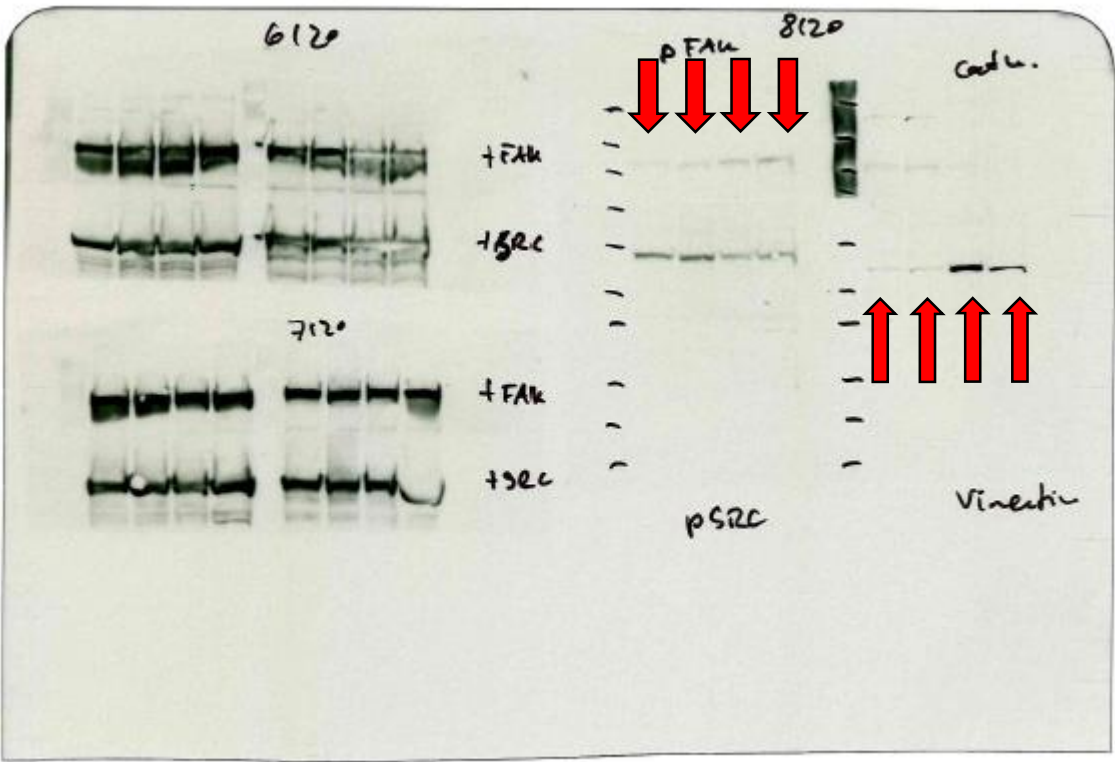

Supplement: Unedited blot and gel images [file jciinsight-9-166402-s104.pdf]
